# The harmonic mean *p*-value for combining dependent tests

**DOI:** 10.1073/pnas.1814092116

**Published:** 2019-01-04

**Authors:** Daniel J. Wilson

**Affiliations:** ^a^Big Data Institute, Nuffield Department of Population Health, Li Ka Shing Centre for Health Information and Discovery, University of Oxford, Oxford OX3 7LF, United Kingdom

**Keywords:** big data, false positives, *p*-values, multiple testing, model averaging

## Abstract

Analysis of “big data” frequently involves statistical comparison of millions of competing hypotheses to discover hidden processes underlying observed patterns of data, for example, in the search for genetic determinants of disease in genome-wide association studies (GWAS). Controlling the familywise error rate (FWER) is considered the strongest protection against false positives but makes it difficult to reach the multiple testing-corrected significance threshold. Here, I introduce the harmonic mean *p*-value (HMP), which controls the FWER while greatly improving statistical power by combining dependent tests using generalized central limit theorem. I show that the HMP effortlessly combines information to detect statistically significant signals among groups of individually nonsignificant hypotheses in examples of a human GWAS for neuroticism and a joint human–pathogen GWAS for hepatitis C viral load. The HMP simultaneously tests all ways to group hypotheses, allowing the smallest groups of hypotheses that retain significance to be sought. The power of the HMP to detect significant hypothesis groups is greater than the power of the Benjamini–Hochberg procedure to detect significant hypotheses, although the latter only controls the weaker false discovery rate (FDR). The HMP has broad implications for the analysis of large datasets, because it enhances the potential for scientific discovery.

## Significance

The widespread use of Bonferroni correction encumbers the scientific process and wastes opportunities for discovery presented by big data, because it discourages exploratory analyses by overpenalizing the total number of statistical tests performed. In this paper, I introduce the harmonic mean *p*-value (HMP), a simple to use and widely applicable alternative to Bonferroni correction motivated by Bayesian model averaging that greatly improves statistical power while maintaining control of the gold standard false positive rate. The HMP has a range of desirable properties and offers a different way to think about large-scale exploratory data analysis in classical statistics.

Analysis of big data has great potential, for instance by transforming our understanding of how genetics influences human disease (1), but it presents unique challenges. One such challenge faces geneticists designing genome-wide association studies (GWAS). Individuals have typically been typed at around 600,000 variants spread across the 3.2 billion base-pair genome. With the rapidly decreasing costs of DNA sequencing, whole-genome sequencing is becoming routine, raising the possibility of detecting associations at ever more variants (2, 3). However, increasing the number of tests of association conventionally requires more stringent *p*-value correction for multiple testing, reducing the probability of detecting any individual association. The idea that analyzing more data may lead to fewer discoveries is counterintuitive and suggests a flaw of logic.

The problem of testing many hypotheses while controlling the appropriate false positive rate is a long-standing issue. The familywise error rate (FWER) is the probability of falsely rejecting a null in favor of an alternative hypothesis in one or more of all tests performed. Controlling the FWER in the presence of some true positives is challenging and considered the strongest form of protection against false positives (4). Unfortunately, the simple and widely used Bonferroni method for controlling the FWER is conservative, especially when the individual tests are positively correlated (5).

Model selection is an important setting affected by correlated tests, in which the same data are used to evaluate many competing alternative hypotheses. Reanalysis of the same outcomes across tests in GWAS causes dependence because of correlations between regressors in different models (6). Other phenomena, such as unmeasured confounders, can induce dependence, even when alternative hypotheses are not mutually exclusive, such as in gene expression analyses (7). The conservative nature of Bonferroni correction, particularly when tests are correlated, exacerbates the stringent criterion of controlling the FWER, jeopardizing sensitivity to detect true signals.

Simulations may be used to identify thresholds that are less stringent yet control the FWER. However, simulating can be time consuming; model-based simulations require knowledge of the dependency structure, which may be limited; and permutation-based procedures are not always appropriate (8).

The false discovery rate (FDR) offers an alternative to the FWER. Controlling the FDR guarantees that, among the significant tests, the proportion in which the null hypothesis is incorrectly rejected in favor of the alternative is limited (9). The widely used Benjamini–Hochberg (BH) procedure (9) for controlling the FDR shares with the Bonferroni method a robustness to positive correlation between tests (10) but is less conservative. These advantages have made FDR a popular alternative to FWER, in practice trading off larger numbers of false positives for more statistical power.

Combined tests offer a different way to improve power. By aggregating multiple hypothesis tests, combined tests are sensitive to signals that may be individually too subtle to detect, especially after multiple testing correction. Their conclusions, therefore, apply collectively rather than to individual tests. Fisher’s method (11) is perhaps the best known and has been widely used in gene set enrichment analysis, but it makes the strong assumption that tests are independent.

Bayesian model averaging offers a way to combine alternative hypotheses in the model selection setting. By comparing groups of alternative hypotheses against a common null, the null hypothesis may be ruled out collectively. In the case of GWAS, even if no individual variant shows sufficient evidence of association in a region, the model-averaged signal across that region may yet achieve sufficiently strong posterior odds (12, 13). Combining tests in this way makes an asset of more data by creating the potential for more fine-grained discovery when the signal is strong enough without the liability of requiring that all hypotheses are evaluated individually at the higher level of statistical stringency.

In this paper, I use Bayesian model averaging to develop a method, the harmonic mean *p*-value (HMP), for combining dependent *p*-values while controlling the strong-sense FWER. The method is derived in the model selection setting and is best interpreted as offering a complementary method to Fisher’s that combines tests by model averaging when they are mutually exclusive, not independent. However, the HMP is applicable beyond model selection problems, because it assumes only that the *p*-values are valid. It enjoys several remarkable properties that offer benefits across a wide range of big data problems.

## Methods

### Model-Averaged Mean Maximum Likelihood.

The original idea motivating this paper was to develop a classical analogue to the model-averaged Bayes factor by deriving the null distribution for the mean maximized likelihood ratio,

$$\bar{R}=\overset{L}{\underset{i=1}{\sum }}w_{i}\;R_{i},$$ [1]

with maximized likelihood ratios $R_{1}\ldots R_{L}$ and weights $w_{1}\ldots w_{L}$, where $\sum_{i=1}^{L}w_{i}=1$.

The maximized likelihood ratio is a classical analogue of the Bayes factor and measures the evidence for the alternative hypothesis $M_{i}$ against the null $M_{0}$ given the data X:

$$R_{i}=\frac{\sup\left\{\mathrm{Pr}\left(X|\theta \right):\theta \in \Theta_{M_{i}}\right\}}{\sup\left\{\mathrm{Pr}\left(X|\theta \right):\theta \in \Theta_{M_{0}}\right\}}.$$

In a likelihood ratio test, the *p*-value is calculated as the probability of obtaining an $R_{i}$ as or more extreme if the null hypothesis were true:

$$p_{i}=\mathrm{Pr}\left(r_{i}\geq R_{i}|\theta \in \Theta_{M_{0}}\right).$$

For nested hypotheses ($\Theta_{M_{0}}\in \Theta_{M_{i}}$), Wilks’ theorem (14) approximates the null distribution of $R_{i}$ as LogGamma(α=ν/2,β=1) when there are ν degrees of freedom.

The distribution of $\bar{R}$ cannot be approximated by central limit theorem, because the LogGamma distribution is heavy tailed, with undefined variance when β≤2. Instead, generalized central limit theorem can be used (15), which states that, for equal weights ($w_{i}=1/L$) and independent and identically distributed $R_{i}$s,

$$R_{1}+\cdots +R_{L}\overset{d}{\to }a_{L}+b_{L}\;R_{\lambda },$$ [2]

where $a_{L}$ and $b_{L}$ are constants and $R_{\lambda }$ is a Stable distribution with tail index λ=β=1. The specific Stable distribution is a type of Landau distribution (16) with parameters that depend on L and ν (*SI Appendix*, section 1). Theory, supported by detailed simulations in *SI Appendix*, section 2, shows that (*i*) the assumptions of equal weights, independence, and identical degrees of freedom can be relaxed and that (*ii*) the Landau distribution approximation performs best when ν=2.

### The Harmonic Mean *p*-Value.

Notably, when ν=2 and the assumptions of Wilks’ theorem are met, the *p*-value equals the inverse maximized likelihood ratio:

$$\begin{array}{ccc} p_{i} & = & \mathrm{Pr}\left(r_{i}\geq R_{i}|\theta \in \Theta_{M_{0}}\right)\\ & = & \mathrm{Pr}\left(\overset{2}{\underset{\nu =2}{\chi }}\geq 2\;\log\;R_{i}\right)=R_{i}^{-1}, \end{array}$$

and therefore, the mean maximized likelihood ratio equals the inverse HMP:

$$\bar{R}=1/\overset{○}{p}.$$ [3]

Under these conditions, interpreting $\bar{R}$ and the HMP is exactly equivalent. This equivalence motivates use of the HMP more generally because of the following.

*i*) The Landau distribution gives an excellent approximation for $\bar{R}$ with ν=2, and hence for $1/\overset{○}{p}$.
*ii*) Wilks’ theorem can be replaced with the simpler assumption that the *p*-values are well calibrated.
*iii*) The HMP will capture similar information to $\bar{R}$ for any degrees of freedom.
*iv*) Combining $p_{i}$s rather than $R_{i}$s automatically accounts for differences in degrees of freedom.

A combined *p*-value, which becomes exact as the number of *p*-values L increases, can be calculated as

$$p_{\overset{○}{p}}=\int_{1/\overset{○}{p}}^{\infty }f_{Landau}\left(x|\log\;L+0.874,\frac{\pi }{2}\right)\;dx,$$ [4]

with the Landau distribution probability density function

$$f_{Landau}\left(x|\mu ,\sigma \right)=\frac{1}{\pi \sigma }\int_{0}^{\infty }e^{-t\frac{\left(x-\mu \right)}{\sigma }-\frac{2}{\pi }t\;log\;t}\;\sin\left(2t\right)\;dt.$$

Remarkably, however, the HMP can be directly interpreted, because it is approximately well calibrated when small. Using the theory of regularly varying functions (see ref. 17),

$$\begin{array}{ccc} p_{\overset{○}{p}} & = & \mathrm{Pr}\left(\overset{L}{\underset{i=1}{\sum }}w_{i}\;p_{i}^{-1}\geq 1/\overset{○}{p}\right)\\ & \approx & \left(\overset{L}{\underset{i=1}{\sum }}w_{i}^{\lambda }\right)\mathrm{Pr}\left(p_{i}^{-1}\geq 1/\overset{○}{p}\right),\overset{○}{p}\to 0\\ & = & \overset{○}{p}. \end{array}$$ [5]

This property suggests the following test, which controls the strong-sense FWER at level approximately α≤0.05 for an HMP ${\overset{○}{p}}_{R}$ calculated on a subset of *p*-values $\left\{p_{i}:i\in R\right\}$:

$$\begin{array}{cc} If\;{\overset{○}{p}}_{R}\leq \alpha \;w_{R}: & \;\text{Reject}M_{0}\text{in favor of}M_{R}\\ Otherwise: & \;\text{Do not reject}M_{0}\text{for}M_{R} \end{array},$$ [6]

where $w_{R}=\sum_{i\in R}w_{i}$. Directly interpreting the HMP using Eq. **6** constitutes a multilevel test in the sense that any significant subset of hypotheses implies that the HMP of the whole set is also significant, because

$$\begin{array}{ccc} If\; & {\overset{○}{p}}_{R} & \leq \alpha \;w_{R}\\ Then & \overset{○}{p} & ={\left(w_{R}\;{\overset{○}{p}}_{R}^{-1}+w_{R^{\prime }}\;{\overset{○}{p}}_{R^{\prime }}^{-1}\right)}^{-1}\\ & & \leq w_{R}^{-1}\;{\overset{○}{p}}_{R}\leq \alpha . \end{array}$$ [7]

Conversely, if the “headline” HMP $\overset{○}{p}$ is not significant, nor is the HMP for any subset ${\overset{○}{p}}_{R}$. The significance thresholds apply no matter how many subsets R are combined and tested.

The above properties show that directly interpreting the HMP (*i*) is a closed testing procedure (4) that controls the strong-sense FWER (*SI Appendix*, section 3); (*ii*) is more powerful than Bonferroni and Simes correction, because the HMP is always smaller than the *p*-values for those tests (*SI Appendix*, section 4); and therefore, (*iii*) produces significant results whenever the Simes-based BH procedure does, although BH only controls the less stringent FDR.

While direct interpretation of the HMP controls the strong-sense FWER, the level at which it does so is only approximately α, and is in fact anticonservative, but only very slightly for small α and small |R|. Assessing the adjusted HMP, ${\overset{○}{p}}_{R}w_{R}^{-1}$, against level $\alpha_{\left|R\right|}$ calculated by inverting Eq. **4** permits a test that is exact up to the order of the Landau distribution approximation (Table 1). (Equivalently, one can compare the exact *p*-value from Eq. **4** with $\alpha \;w_{R}$.) Simulations suggest that this exact test remains more powerful than Bonferroni, Simes, and therefore, BH (*SI Appendix*, section 4).

**Table 1.** Significance thresholds $\alpha_{\left|R\right|}$ for ${\overset{○}{p}}_{R}w_{R}^{-1}$, the adjusted HMP, for varying numbers of alternative hypotheses |R| and false positive rates α

|R|	α=0.05	α=0.01	α=0.001
10	0.040	0.0094	0.00099
100	0.036	0.0092	0.00099
1,000	0.034	0.0090	0.00099
10,000	0.031	0.0088	0.00098
100,000	0.029	0.0086	0.00098
1,000,000	0.027	0.0084	0.00098
10,000,000	0.026	0.0083	0.00098
100,000,000	0.024	0.0081	0.00098
1,000,000,000	0.023	0.0080	0.00097

I recommend the use of this asymptotically exact test, available in the R package “harmonicmeanp” (https://CRAN.R-project.org/package=harmonicmeanp), on which all subsequent analyses in *Results* are based. Analyses based on direct interpretation of the HMP are also presented and reveal the practical differences between the approaches to be small for α=0.05.

### Choice of Weights.

I anticipate that the HMP will usually be used with equal weights, as are procedures such as Bonferroni correction and Simes’ test. *SI Appendix*, section 5 considers optimal weights. Based on Bayesian (18) and classical arguments and assuming that all tests have good power, the optimal weight $w_{i}$ is found to be proportional to the product of the prior probability of alternative hypothesis $M_{i}$ and the expectation of $p_{i}$ under $M_{i}$. This optimal weighting would favor alternatives that are more probable a priori while penalizing those associated with more powerful tests.

Consequently, the use of equal weights can be interpreted as assuming that all alternative hypotheses are equally likely a priori and that all tests are equally powerful. If tests are not equally powerful for a given “effect size,” the equal power assumption implies that alternatives associated with inherently less powerful tests are expected to have larger effect sizes a priori, a testable assumption that has been used often in GWAS (19).

## Results

The main result of this paper is that the weighted harmonic mean *p*-value of any subset R of the *p*-values $p_{1}\ldots p_{L}$,

$${\overset{○}{p}}_{R}=\frac{\sum_{i\in R}w_{i}}{\sum_{i\in R}w_{i}/p_{i}},$$ [8]

(*i*) combines the evidence in favor of the group of alternative hypotheses R, (*ii*) is an approximately well-calibrated *p*-value for small values, and (*iii*) controls the strong-sense FWER at level approximately α≤0.05 when compared against the threshold $\alpha \;w_{R}$, no matter how many other subsets of the same *p*-values are tested ($w_{R}=\sum_{i\in R}w_{i}$ and $\sum_{i=1}^{L}w_{i}=1$). An asymptotically exact test is also available (Eq. **4**). The HMP has several helpful properties that arise from generalized central limit theorem. It is:

*i*) Robust to positive dependency between *p*-values.
*ii*) Insensitive to the exact number of tests.
*iii*) Robust to the distribution of weights w.
*iv*) Most influenced by the smallest *p*-values.

The HMP outperforms Bonferroni and Simes (5) correction. This advantage over Simes’ test means that whenever the BH procedure (9), which controls only the FDR, finds significant hypotheses, the HMP will find significant hypotheses or groups of hypotheses. The HMP complements Fisher’s method for combining independent *p*-values (11), because the HMP is more appropriate when (*i*) rejecting the null implies that only one alternative hypothesis may be true and not all of them or (*ii*) the *p*-values might be positively correlated and cannot be assumed to be independent.

### HMP Enables Adaptive Multiple Testing Correction by Combining *p*-Values.

That the Bonferroni method for controlling the FWER can be overly stringent, especially when the tests are nonindependent, has long been recognized. In Bonferroni correction, a *p*-value is deemed significant if p≤α/L, which becomes more stringent as the number of tests L increases. Since human GWAS began routinely testing millions of variants by statistically imputing untyped variants, a new convention was adopted in which a *p*-value is deemed significant if $p\leq 5\times 10^{-8}$, a rule that implies that the effective number of tests is no more than $L=10^{6}$. Several lines of argument were used to justify this threshold (20–22), most applicable specifically to human GWAS.

In contrast, the HMP affords strong control of the FWER while avoiding both simulation studies and the undue stringency of Bonferroni correction, an advantage that increases when tests are nonindependent. To show how the HMP can recover significant associations among groups of tests that are individually nonsignificant, I reanalyzed a GWAS of neuroticism (23), defined as a tendency toward intense or frequent negative emotions and thoughts (24). Genotypes were imputed for L=6 524 432 variants across 170 911 individuals. I used the HMP to perform model-averaged tests of association between neuroticism and variants within contiguous regions of 10 kb, 100 kb, 1,000 kb, 10 Mb, entire chromosomes, and the whole genome, assuming equal weights across variants (*SI Appendix*, section 6).

**Fig. 1.**
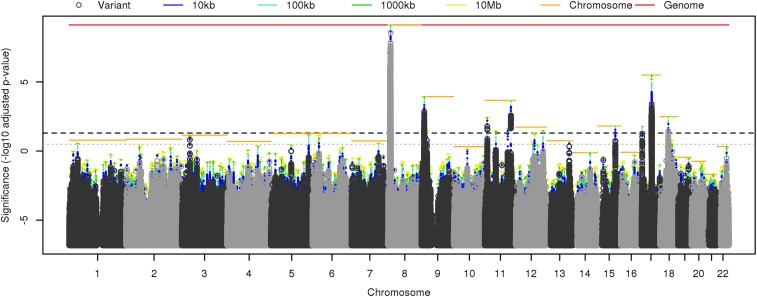
Results of a GWAS of neuroticism in 170,911 people (23). This Manhattan plot shows the significance of the association between neuroticism and L=6 524 432 variants (dark and light gray points) and overlapping regions of lengths 10 kb (blue bars), 100 kb (cyan bars), 1,000 kb (green bars), 10,000 kb (yellow bars), entire chromosomes (orange bars), and the whole genome (red bar). Significance is defined as the $-\log_{10}$ adjusted *p*-value, where the *p*-value for region R is defined by Eq. **4** and adjusted by a factor $w_{R}^{-1}$ to enable direct comparison with the threshold α=0.05 (black dashed line). The conventional threshold of $\alpha =\left(5\times 10^{-8}\right)L$ is shown for comparison (gray dotted line).

Fig. 1 shows the *p*-value from Eq. **4** for each region R adjusted by a factor $w_{R}^{-1}$ to enable direct comparison with the significance threshold α=0.05. Similar results were obtained from direct interpretation of the HMP (*SI Appendix*, Fig. S1). Model averaging tends to make significant and near-significant adjusted *p*-values more significant. For example, for every variant significant after Bonferroni correction, the model-averaged *p*-value for the corresponding chromosome was found to be at least as significant.

Model averaging increases significance more when combining a group of comparably significant *p*-values, e.g., the top hits in chromosome 9. The least improvement is seen when one *p*-value is much more significant than the others, e.g., the top hit in chromosome 3. This behavior is predicted by the tendency of harmonic means to be dominated by the smallest values. In the extreme case that one *p*-value dominates the significance of all others, the HMP test becomes equivalent to Bonferroni correction. This implies that Bonferroni correction might not be improved on for “needle-in-a-haystack” problems. Conversely, dependency among tests actually improves the sensitivity of the HMP, because one significant test may be accompanied by other correlated tests that collectively reduce the harmonic mean *p*-value.

In some cases, the HMP found significant regions where none of the individual variants were significant. For example, no variants on chromosome 12 were significant by Bonferroni correction nor by the conventional genome-wide significance threshold of $5\times 10^{-8}$. However, the HMP found significant 10-Mb regions spanning several peaks of nonsignificant individual *p*-values. One of those, variant rs7973260, which showed an individual *p*-value for association with neuroticism of $2.4\times 10^{-7}$, had been reported as also associated with depressive symptoms ($p=1.8\times 10^{-9}$). Such cross-association or “quasireplication,” in which a variant is nearly significant for the trait of interest and significant for a related trait, can be regarded as providing additional support for the variant’s involvement in the trait of interest (23).

In chromosome 3, individual variants were found to be significant by the conventional threshold of $5\times 10^{-8}$, but neither Bonferroni correction nor the HMP agreed that those variants or regions were significant at an FWER of α=0.05. Indeed, the HMP found chromosome 3 nonsignificant as a whole. Variant rs35688236, which had the smallest *p*-value on chromosome 3 of $2.4\times 10^{-8}$, had not validated when tested in a quasireplication exercise that involved testing variants associated with neuroticism for association with subjective wellbeing or depressive symptoms (23).

These observations illustrate that the HMP adaptively combines information among groups of similarly significant tests where possible, while leaving lone significant tests subject to Bonferroni-like stringency, providing a general approach to combining *p*-values that does not require specific knowledge of the dependency structure between tests.

### HMP Allows Large-Scale Testing for Higher-Order Interactions Without Punitive Thresholds.

Scientific discovery is currently hindered by avoidance of large-scale exploratory hypothesis testing for fear of attracting multiple testing correction thresholds that render signals found by more limited testing no longer significant. A good example is the approach to testing for pairwise or higher-order interactions between variants in GWAS. The Bonferroni threshold for testing all pairwise interactions invites a threshold (L+1)/2 times more stringent than the threshold for testing variants individually, and strictly speaking this must be applied to every test, even though this is highly conservative because of the dependency between tests. The alternative of controlling the FDR risks a high probability of falsely detecting artifacts among any genuine associations discovered. Therefore, interactions are not usually tested for.

To show how model averaging using the HMP greatly alleviates this problem, I reanalyzed human and pathogen genetic variants from a GWAS of pretreatment viral load in hepatitis C virus (HCV)-infected patients (25) (*SI Appendix*, section 7). Jointly analyzing the influence of human and pathogen variation on infection is an area of great interest, but it requires a Bonferroni threshold of $\alpha /\left(L_{H}\;L_{P}\right)$ when there are $L_{H}$ and $L_{P}$ variants in the human and pathogen genomes, respectively, compared with $\alpha /\left(L_{H}+L_{P}\right)$ if testing the human and pathogen variants separately. In this example, $L_{H}=399\;420$ and $L_{P}=827$.

**Fig. 2.**
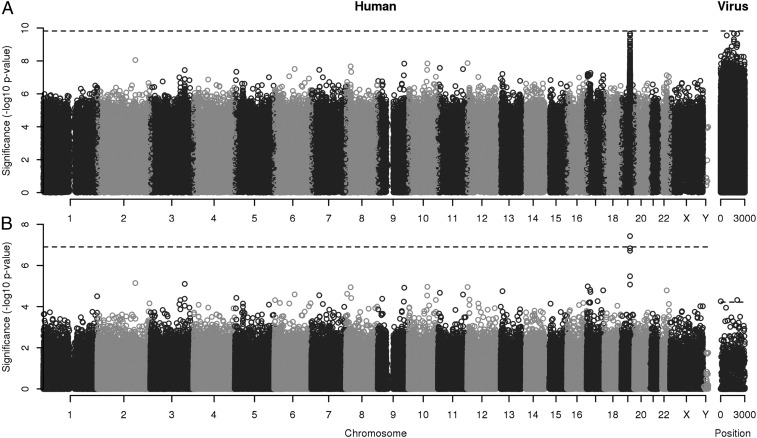
Joint human–pathogen GWAS reanalysis of viral load in 410 HCV genotype 3a-infected white Europeans (25). All pairs of human nucleotide variants and viral amino acid variants were tested for association. Interactions between human and virus variants’ effects on viral load were not constrained to be additive. (*A*) Significance of 330,320,340 tests plotted by position of both the human and the viral variants. (*B*) Significance of 399,420 human variants model averaged using the HMP over every possible interaction with 827 viral variants and vice versa. The significance thresholds controlling the FWER at α=0.05 are indicated (black dashed lines): $\alpha /\left(L_{H}L_{P}\right)$, $\alpha /L_{H}$, and $\alpha /L_{P}$.

In the original study, a known association with viral load was replicated at human chromosome 19 variant rs12979860 in *IFNL4* ($p=5.9\times 10^{-10}$), below the Bonferroni threshold of $1.3\times 10^{-7}$ for 399 420 tests. The most significant pairwise interaction that I found, assuming equal weights, involved the adjacent variant rs8099917 with $p=2.2\times 10^{-10}$. However, this did not meet the more stringent Bonferroni threshold of $1.5\times 10^{-10}$ for 330 million tests (Fig. 2*A*). If the original study’s authors had performed and reported 330 million tests, they could have been compelled to declare the marginal association in *IFNL4* nonsignificant, despite what intuitively seems like a clear signal.

Model averaging using the HMP reduces this disincentive to perform additional related tests. Fig. 2*B* shows that, despite no significant pairwise tests involving rs8099917, model averaging recovered a combined *p*-value of $3.7\times 10^{-8}$, below the multiple testing threshold of $1.3\times 10^{-7}$ for the 399 420 model-averaged tests. Additionally, two viral variants produced statistically significant model-averaged *p*-values of $5.5\times 10^{-5}$ and $4.8\times 10^{-5}$ at polyprotein positions 10 and 2,061 in the capsid and NS5a zinc finger domain (GenBank accession no. AQW44528), below the multiple testing threshold of $6.0\times 10^{-5}$ for the 827 model-averaged tests.

These results show how model averaging using the HMP can assist discovery making by (*i*) encouraging tests for higher-order interactions when they otherwise would not be attempted and (*ii*) recovering lost signals of marginal associations after performing an “excessive” number of tests.

### Untangling the Signals Driving Significant Model-Averaged *p*-Values.

When more than one alternative hypothesis is found to be significant, either individually or as part of a group, it is desirable to quantify the relative strength of evidence in favor of the competing alternatives. This is particularly true when disentangling the contributions of a group of individually nonsignificant alternatives that are significant only in combination.

Sellke et al. (18) proposed a conversion from *p*-values to Bayes factors which, when combined with prior information and test power through the model weights, produces posterior model probabilities and credible sets of alternative hypotheses. *SI Appendix*, section 5 details how the Bayes factors are approximately proportional to the weighted inverse *p*-values. This linearity mirrors the HMP itself, the inverse of which is an arithmetic mean of the inverse *p*-values.

**Fig. 3.**
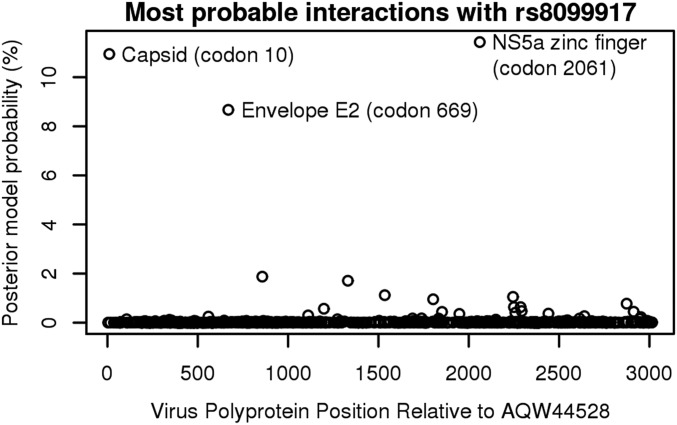
In the joint human–HCV GWAS, the approximate posterior probability of association with rs8099917 was 54.4% in total, with the most probable interactions involving three polyprotein positions.

After conditioning on rejection of the null hypothesis by normalizing the approximate model probabilities to sum to 100%, the probability that the association involved human variant rs8099917 was 54.4%. This signal was driven primarily by the three viral variants with the highest probability of interacting with rs8099917 in their effect on pretreatment viral load: position 10 in the capsid (10.9%), position 669 in the E2 envelope (8.7%), and position 2,061 in the NS5a zinc finger domain (11.4%) (Fig. 3). Even though the model-averaged *p*-value for the envelope variant was not itself significant, this revealed a plausible interaction between it and the most significant human variant rs8099917.

## Discussion

The HMP provides a way to calculate model-averaged *p*-values, providing a powerful and general method for combining tests while controlling the strong-sense FWER. It provides an alternative to both the overly conservative Bonferroni control of the FWER, and the lower stringency of FDR control. The HMP allows the incorporation of prior information through model weights and is robust to positive dependency between the *p*-values. The HMP is approximately well calibrated for small values, while a null distribution, derived from generalized central limit theorem, is easily computed. When the HMP is not significant, neither is any subset of the constituent tests.

The HMP is more appropriate for combining *p*-values than Fisher’s method when the alternative hypotheses are mutually exclusive, as in model comparison. When the alternative hypotheses all have the same nested null hypothesis, the HMP is interpreted in terms of a model-averaged likelihood ratio test. However, the HMP can be used more generally to combine tests that are not necessarily mutually exclusive but that may have positive dependency, with the caveat that more powerful approaches may be available depending on the context. The HMP can be used alone or in combination: for example, with Fisher’s method to combine model-averaged *p*-values between groups of independent data.

The theory underlying the HMP provides a fundamentally different way to think about controlling the FWER through multiple testing correction. The Bonferroni threshold increases linearly with the number of tests, whereas the HMP is the reciprocal of the mean of the inverse *p*-values. To maintain significance with Bonferroni correction, the minimum *p*-value must decrease linearly as the number of tests increases. This strongly penalizes exploratory and follow-up analyses. In contrast, when the false positive rate α is small, maintenance of significance with the HMP requires only that the mean inverse *p*-value remains constant as the number of tests increases. This does not penalize exploratory and follow-up analyses so long as the “quality” of the additional hypotheses tested, measured by the inverse *p*-value, does not decline.

Through example applications to GWAS, I have shown that the HMP combines tests adaptively, producing Bonferroni-like adjusted *p*-values for needle-in-a-haystack problems when one test dominates, but able to capitalize on numerous strongly significant tests to produce smaller adjusted *p*-values when warranted. I have shown how model averaging using the HMP encourages exploratory analysis and can recover signals of significance among groups of individually nonsignificant tests, properties that have the potential to enhance the scientific discovery process.

## Supplementary Material

Supplementary File
