# Supplementary material for: The harmonic mean p-value for combining dependent tests
Source: Proc Natl Acad Sci U S A. 2019 Jan 4;116(4):1195–200. doi: 10.1073/pnas.1814092116 (PMC6347718; doi:10.1073/pnas.1814092116)
Supplement: Supplementary File [file pnas.1814092116.sapp.pdf]

The harmonic mean p-value for combining dependent tests

Daniel J. Wilson

Supporting Information (SI)

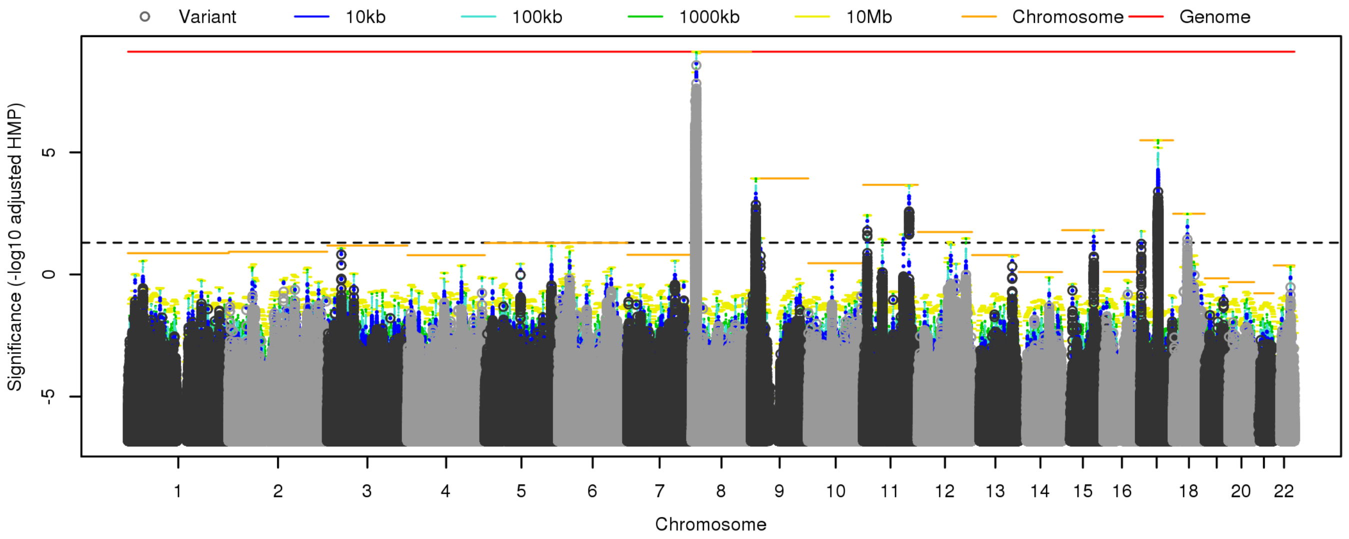

Fig. S1. Results of a GWAS of neuroticism in 170 911 people. This Manhattan plot shows the significance of association between neuroticism and $L = 6\,524\,432$ variants (dark and light grey points) and overlapping regions of length 10, 100, 1 000 and 10 000 kb (blue, cyan, green and yellow bars), entire chromosomes (orange bars) and the whole genome (red bar). Significance is defined as the $-\log_{10}$ adjusted harmonic mean p-value, where the HMP for region $\mathcal{R}$ is defined by Equation 8, and adjusted by a factor $w_{\mathcal{R}}^{-1}$ to enable direct comparison to the threshold $\alpha = 0.05$ (black dashed line). The HMP is approximately well-calibrated for small values, e.g. below 0.05.

Supplementary Methods

Contents

1 Derivation of the null distributions for the model-averaged mean maximized likelihood ratio and harmonic mean p-value **2**

A Generalized central limit theorem approximates the distribution of the mean maximized likelihood ratio 2

B Parameterization of the Stable distribution approximation for $\nu = 2$ 3

C Parameterization of the Stable distribution approximation for $\nu \neq 2$ 4

2 The null distributions of MAMML and the HMP are robust to the number of tests, unequal weights, unequal degrees of freedom and dependency between the tests **4**

A Performance of the approximation under favourable assumptions 5

B Robustness of the approximation to unequal prior model weights 6

C Robustness of the approximation to extreme dependency between tests 7

D Robustness of the approximation to unequal degrees of freedom among tests 7

E Performance of the harmonic mean p-value 8

3 Controlling the strong-sense family-wise error rate of the HMP test **9**

A Derivation of the closed testing procedure 10

B Bonferroni achieves strong-sense FWER 10

C A multilevel CTP for the HMP 10

4 The relationship between the HMP and other combined tests **11**
A The HMP is closely related to but more powerful than Simes' procedure 11
B Complementarity to Fisher's method for combining p-values 12
C Position within Loughin's classification of methods for combining p-values 14

5 Bayesian connections and comparing competing alternative hypotheses **14**
A Similarity between Bayesian model-averaged significance testing and the HMP 14
B Parallels between Bayesian model-averaging and Simes' method 15
C Interpretation of the harmonic mean p-value as a Bayesian procedure 16
D Relaxing significance thresholds when multiple alternative hypotheses are true 20

6 GWAS of neuroticism **21**

7 GWAS of HCV pre-treatment viral load **21**

8 SI references **21**

1. Derivation of the null distributions for the model-averaged mean maximized likelihood ratio and harmonic mean p-value

A. Generalized central limit theorem approximates the distribution of the mean maximized likelihood ratio. The motivating idea of the paper was to develop a classical model-averaged mean maximum likelihood (MAMML), analogous to the model-averaged Bayes factor, by seeking a null distribution for the mean maximized likelihood ratio defined in Equation 1 as

$$\bar{R} \quad = \quad \sum_{i=1}^{L} w_i \, R_i.$$

When the assumptions of Wilks' theorem (1) are met, the null distribution of the maximized likelihood ratio

$$R_i \quad \sim \quad \text{LogGamma}\left(\alpha = \frac{\nu}{2}, \beta = 1\right) \qquad [9]$$

where α and β are the shape and rate parameters of the LogGamma distribution and ν is the difference in the number of parameters between the alternative and nested null hypotheses. The LogGamma distribution has probability density function

$$f_{\text{LogGamma}}(x \,|\, \alpha, \beta) \quad = \quad \frac{\beta^\alpha}{\Gamma(\alpha)} \log(x)^{\alpha-1} \, x^{-(\beta+1)}. \qquad [10]$$

When $\nu = 2$, interpreting the mean maximized likelihood ratio is equivalent to interpreting the harmonic mean p-value (HMP) because, from Equation 3, $\bar{R} = 1/\mathring{p}$. This means that the results for $\bar{R}$ when $\nu = 2$ also apply to $1/\mathring{p}$, except that the assumptions of Wilks' theorem no longer need to hold, only that the individual p-values follow the uniform distribution under the null hypothesis, and therefore that $1/p_i$ follows a $\text{LogGamma}(1,1)$ distribution. In what follows, the derivation for $1/\mathring{p}$ is therefore the same as for $\bar{R}$ with $\nu = 2$.

Central limit theorem cannot be used to approximate the null distribution of $\bar{R}$ (or $1/\mathring{p}$) because the mean and variance of the LogGamma distribution are undefined for $\beta \leq 1$ and $\beta \leq 2$ respectively. However, when the R_is are independent and identically distributed with regularly varying cumulative distribution function, generalized central limit theorem (2) states that

$$R_1 + \cdots + R_L \xrightarrow{d} a_L + b_L \, R_\lambda \qquad [11]$$

where a_L and b_L are constants, λ is the heavy-tail index of the R_is and R_λ is a Stable distribution with tail index λ. The LogGamma distribution can be shown to be regularly varying, and its cumulative distribution function can be approximated in order to apply generalized central limit theorem. A positive measurable function $f(x)$ is defined to be regularly varying (3, 4) if

$$\lim_{x\to\infty} \frac{f(tx)}{f(x)} = t^{-\lambda} \qquad \text{for all } t > 0, \qquad [12]$$

and slowly varying if $\lambda = 0$. Any regularly varying function $f(x)$ can be written in terms of a slowly varying function $S(x)$ as

$$f(x) = S(x)\, x^{-\lambda}. \qquad [13]$$

The probability density function of the LogGamma distribution is regularly varying with heavy-tail index $\lambda = \beta + 1$ and slowly varying function

$$S_{\text{LogGamma}}(x) = \frac{\beta^{\alpha}}{\Gamma(\alpha)} \log(x)^{\alpha-1}. \qquad [14]$$

The cumulative distribution function of the LogGamma distribution is thus regularly varying with a *different* heavy-tail index $\lambda = \beta$ because, by Karamata's theoreom (see e.g. (4)),

$$\int_x^{\infty} S(u)\, u^{-(\lambda+1)} \mathrm{d}u \quad \approx \quad \frac{S(x)\, x^{-\lambda}}{\lambda}, \qquad x \to \infty, \qquad [15]$$

for $\lambda > 0$, so it can be approximated by

$$\begin{aligned} F_{\text{LogGamma}}(x \,|\, \alpha, \beta) \quad &= \quad 1 - \int_x^{\infty} f_{\text{LogGamma}}(u \,|\, \alpha, \beta)\, \mathrm{d}u \\ &\approx \quad 1 - \frac{\beta^{\alpha-1}}{\Gamma(\alpha)} \log(x)^{\alpha-1}\, x^{-\beta}, \qquad x \to \infty. \end{aligned} \qquad [16]$$

B. Parameterization of the Stable distribution approximation for $\nu = 2$. To utilize generalized central limit theorem it is necessary to find the parameters of the Stable distribution R_λ, the location constant a_L and the scale constant b_L. This can be achieved (2) by approximating the cumulative distribution function of the R_is as

$$\begin{aligned} 1 - F_R(x) \quad &\approx \quad c\, x^{-\lambda}, \qquad x \to \infty, \\ F_R(-x) \quad &\approx \quad d\, x^{-\lambda}, \qquad x \to \infty. \end{aligned} \qquad [17]$$

The Stable distribution R_λ then has tail index parameter λ and skewness parameter $(c-d)/(c+d)$. Since a LogGamma distributed random variable cannot take a value less than one implies that $d = 0$, so the skewness parameter takes its maximum value of one, producing an Extremal Stable distribution. When $\nu = 2$, the LogGamma$(\nu/2, 1)$ distribution for the R_is simplifies to a Pareto distribution with tail distribution function

$$1 - F_R(x) \quad = \quad x^{-\lambda}, \qquad [18]$$

and the representation in Equation 17 becomes exact. So for $\nu = 2$, $c = 1$. When $\lambda = 1$, as here because λ equals the rate parameter β of the LogGamma distribution, (5) defines the location constant to be (with a less precise form in (2)):

$$a_L \quad = \quad (c - d)\, L \left(\log L + 1 - C_\gamma - \log \frac{2}{\pi} \right), \qquad [19]$$

where $C_\gamma \approx 0.5772157$ is the Euler-Mascheroni constant, and the scale constant to be (2):

$$b_L \quad = \quad \frac{\pi}{2} (c + d)\, L. \qquad [20]$$

So $\bar{R}$ is said to follow an Extremal Stable distribution which, in Nolan's S0 parameterization (6) of the Stable distribution, $\mathrm{S}(\alpha, \beta, \gamma, \delta; 0)$, can be written as

$$\bar{R} \sim \mathrm{S}\left(\lambda, \frac{c-d}{c+d}, \frac{b_L}{L}, \frac{a_L}{L}; 0\right) \sim \mathrm{S}\left(1, 1, c\,\pi/2, c\,(\log L + 0.874367); 0\right). \qquad [21]$$

Therefore the combined p-value for the mean maximized likelihood ratio is

$$p_{\bar{R}} \quad = \quad 1 - F_{\text{Stable}}\left(\bar{R} \,|\, 1, 1, c\,\pi/2, c\,(\log L + 0.874367); 0\right) \qquad [22]$$

which can be computed via the pEstable function of the FMStable R package (7) as

```
1 - pEstable(Rbar, setParam(alpha = 1, location = c * (log(L) + 0.874367), logscale = log(c * pi/2), pm = 0))
```

An explicit form for the probability density function of $\bar{R}$ can be obtained by noting the equivalence of the Extremal Stable distribution with heavy-tail index $\lambda = 1$ and the Landau distribution (8, 9), as can be seen from a comparison of the characteristic functions (10, 11). In Nolan's notation, the characteristic function of the Stable $\mathrm{S}(1, 1, \gamma, \delta; 0)$ distribution (6) is

$$\mathrm{E}\exp\left\{iu\bar{R}\right\} \quad = \quad \exp\left\{iu\delta - |u\,\gamma|\left[1 + \frac{2}{\pi}i\log|u\,\gamma|\mathrm{sign}(u)\right]\right\} \qquad [23]$$

and the characteristic function for a two-parameter Landau(μ, σ) distribution can be written identically but with location parameter $\mu = \delta$ and scale parameter $\sigma = \gamma$. (Landau's original distribution, which describes the random loss of energy of fast charged particles as they cross a thin layer, is retrieved under this parameterization when $\mu = \log(\pi/2)$ and $\sigma = \pi/2$.) Expressed as a Landau distribution,

$$\bar{R} \sim \mathrm{Landau}\left(\mu = \frac{a_L}{L}, \sigma = \frac{b_L}{L}\right) \sim \mathrm{Landau}\left(\mu = c\,(\log L + 0.874367)\,, \sigma = c\,\frac{\pi}{2}\right). \qquad [24]$$

The advantage of this form is that the probability density function can be written explicitly (12) as

$$f_{\mathrm{Landau}}(x\,|\,\mu, \sigma) \quad = \quad \frac{1}{\pi\sigma}\int_0^\infty \mathrm{e}^{-t\frac{(x-\mu)}{\sigma} - \frac{2}{\pi}t\log t}\,\sin(2t)\,\mathrm{d}t \qquad [25]$$

which means that the combined p-value can be computed numerically without the specialist package, if necessary, as

$$p_{\bar{R}} \quad = \quad \int_{\bar{R}}^\infty f_{\mathrm{Landau}}\left(x\,|\,c\,(\log L + 0.874367)\,, c\,\frac{\pi}{2}\right)\mathrm{d}x \qquad [26]$$

and likewise for the HMP, substituting $\bar{R} = 1/\overset{\circ}{p}$ and $c = 1$, as per Equation 4.

C. Parameterization of the Stable distribution approximation for $\nu \neq 2$. When $\nu \neq 2$, the LogGamma$(\nu/2, 1)$ distribution no longer simplifies to a Pareto distribution, and the representation of Equation 17 is no longer exact. This means that

$$c \quad = \quad x^\lambda\,(1 - F_R(x))\,, \qquad [27]$$

is no longer constant with respect to x, so the choice will affect the performance of the Stable distribution approximation. Since the null distribution of the maximized likelihood ratios is heavy tailed, the mean is likely to be dominated by the largest value, i.e. $R_{(L)} \approx L\,\bar{R}$, where $R_{(i)}$ is the ith largest value. Conversely, if every ratio contributed equally to the mean, all values would be $R_i = \bar{R}$. These observations suggest that the relevant value of x at which to evaluate c lies between $\bar{R}$ and $L\,\bar{R}$, with the upper end of the range of most relevance. For simplicity I investigated the performance of the Stable distribution approximation at both endpoints of the range, defining with $\lambda = 1$,

$$c_1(\bar{R}) \quad = \quad \bar{R}\left(1 - F_R(\bar{R})\right), \qquad \text{and} \qquad [28]$$

$$c_L(\bar{R}) \quad = \quad (L\,\bar{R})\left(1 - F_R(L\,\bar{R})\right). \qquad [29]$$

The combined p-value for the mean maximized likelihood ratio is approximated as per Equation 22 but with $c = c_1(\bar{R})$ or $c = c_L(\bar{R})$ for the two approximations. This can be computed for example in the FMStable R package (7) using

```
c = Rbar * (1 - pgamma(log(Rbar), nu/2, 1))    or    c = L * Rbar * (1 - pgamma(log(L * Rbar), nu/2, 1))
```

or it can be computed using a general purpose numerical integration tool via the Landau distribution in Equation 26. The next section shows that c_1 should be used when $\nu < 2$ and c_L should be used when $\nu > 2$.

2. The null distributions of MAMML and the HMP are robust to the number of tests, unequal weights, unequal degrees of freedom and dependency between the tests

The derivation above assumes equal prior weights for the alternative hypotheses, equal degrees of freedom and independence between the tests. However, generalized central limit theorem is robust to departures from these assumptions. I investigated the performance of the Stable distribution approximation across a range of scenarios. These simulations demonstrated key properties of the null distribution of $\bar{R}$ as the mean of a large number of regularly varying random variables (the R_is):

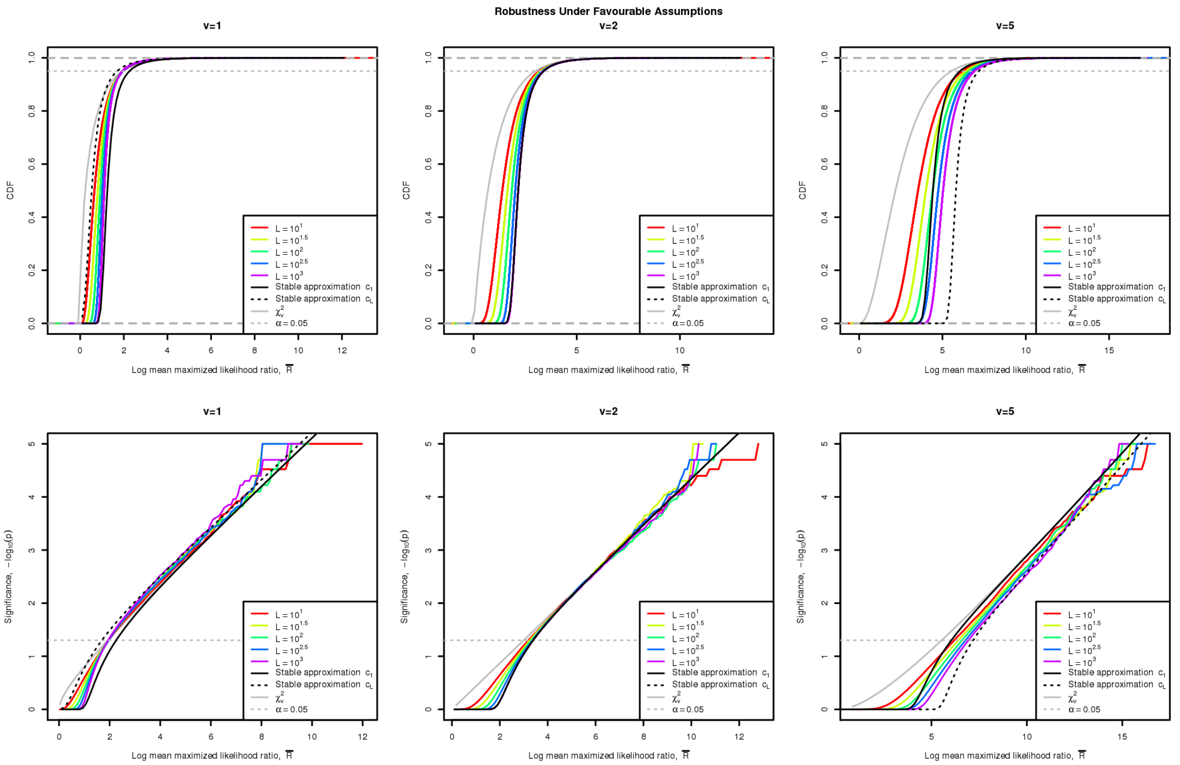

Fig. S2. Robustness of the Stable approximation under favourable assumptions. Cumulative distribution functions (top panels) and 'significance' (i.e. $-\log_{10}$ tail probabilities, bottom panels) of the mean maximized likelihood ratio, $\bar{R}$, are shown across three degrees of freedom ($\nu = 1, 2, 5$) comparing 100,000 simulations (coloured lines) to analytical approximations based on the Stable distribution (black lines). Two Stable distribution approximations (c_1, Equation 28 and c_L, Equation 29) were calculated assuming $L = 10^3$ tests, whereas the simulations were performed with a variable number of tests comprising $L = 10^1, 10^{1.5}, 10^2, 10^{2.5}, 10^3$. The simulations made assumptions favourable to the Stable distribution approximation: independence between tests and equal weights. For comparison, a χ^2_ν distribution is also shown (grey line).

1. The upper tail of the null distribution of $\bar{R}$ behaves like that of an individual test R_i
2. The null distribution of $\bar{R}$ is fairly insensitive to the number of tests, L
3. The null distribution of $\bar{R}$ is insensitive to the model weights
4. The null distribution of $\bar{R}$ is insensitive to positive dependency between the R_is
5. When the R_is have differing degrees of freedom, those with the largest dominate the null distribution of $\bar{R}$

In particular, the simulations demonstrated the superior performance of the approximation when $\nu = 2$. This superior performance, and the equivalence between the mean maximized likelihood ratio when $\nu = 2$ with the intuitive representation as a harmonic mean p-value, motivated the focus on the HMP in the rest of the paper.

A. Performance of the approximation under favourable assumptions. Property 1, that the upper tail of $\bar{R}$ behaves like the upper tail of the R_is, is an inherent property of generalized central limit theorem (4), and is expressed by the approximation in Equation 5. Property 2, an insensitivity to the exact number of tests, L, is revealed by the parameterization of the Stable distribution approximation for the null distribution of $\bar{R}$ (Equations 21 and 22). For $\nu = 2$ degrees of freedom, when the scaling of the tail probability c equals one, the location parameter of the Stable distribution increases in proportion to $\log L$, i.e. more slowly than linearly, and the scale parameter does not change at all in relation to L. The heavy-tail index and skewness parameters are also unaffected by L regardless of ν. When $\nu \neq 2$, the scale for the tail probability, c, is only approximately locally constant with respect to $\bar{R}$, and may depend on L. This weak dependency on L is represented in the c_L approximation of the scale for the tail probability (Equation 29).

The simulations demonstrated these properties empirically. I performed 100 000 simulations each of $L = 10^1$, $10^{1.5}$, 10^2, $10^{2.5}$ and 10^3 independent maximized likelihood ratio test statistics, $\{R_i, i = 1 \ldots L\}$ and for each simulation calculated $\bar{R}$. I repeated the simulations for three degrees of freedom: $\nu = 1$, 2 and 5. The rainbow-coloured lines in Figure S2 show the empirical cumulative distribution functions (top panels) and empirical 'significance' or $-\log_{10}$ tail probabilities (bottom panels) from $L = 10^1$ (red) to $L = 10^3$ (purple).

The top panels in Figure S2 show that the total number of tests, L, has a substantial effect on the null distribution of $\bar{R}$ below the 95th percentile (indicated by the grey horizontal dashed line labelled $\alpha = 0.05$). In contrast, the

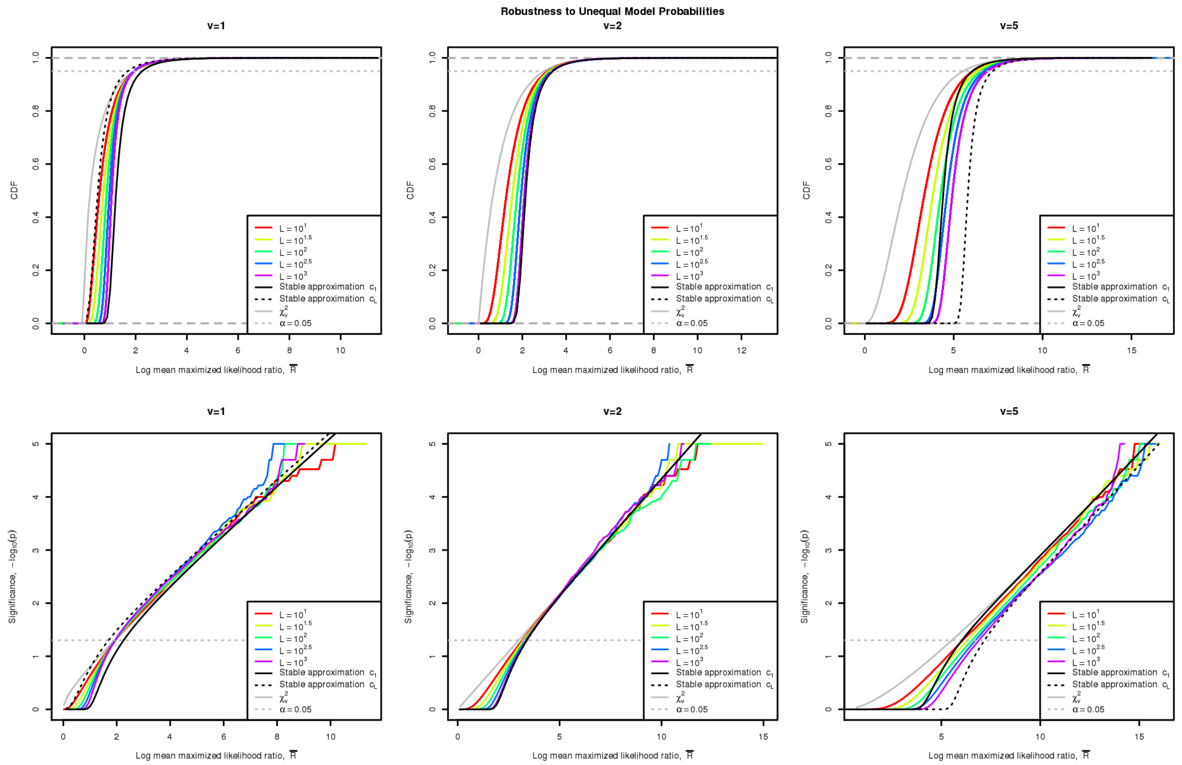

Fig. S3. Robustness of the Stable approximation to unequal prior model probabilities, i.e. weights. The simulations were conducted as for Figure S2 except that model weights were used in the calculation of $\bar{R}$. These weights were also simulated, independently for every simulation, from a symmetric Dirichlet distribution with parameter vector $\mathbf{1}_L$. This is equivalent to simulating each individual weight from a Beta$(1, L-1)$ distribution. So the mean and variance of the weight per test were $1/L$ and $(L-1)/L^2/(L+1)$.

bottom panels show that the upper tails of the distribution above the 95th percentile are almost identical irrespective of L, and converge to the distribution of an individual R_i (grey solid lines), demonstrating Properties 1 and 2. These observations are consistent with much slower convergence of the left tail of the distribution to the Stable distribution limit. Nevertheless, the utility of the null distribution approximation is to calculate p-values, i.e. upper tail probabilities, for which the simulations demonstrate the desired insensitivity of the upper tail to L.

As anticipated, the insensitivity to L is superior for $\nu = 2$. Notably, the direction of the effect of L on the relative magnitude of the significance ($-\log_{10}$ tail probabilities) switches for $\nu < 2$ and $\nu > 2$. For $\nu = 1$, the significance of $\bar{R}$ is greater for larger L. For $\nu = 5$, the significance of $\bar{R}$ is greater for smaller L. This behaviour has an important effect on whether the Stable distribution approximation is conservative or anti-conservative when $\nu \neq 2$.

The Stable distribution approximations c_1 and c_L for the null distribution of $\bar{R}$ (Equations 28 and 29) are shown by the solid and dashed black lines respectively. Both approximations were calculated only for the maximum value of $L = 10^3$ to give an indication of the robustness of the tests to model misspecification, in particular over-estimation of the number of independent tests. For $\nu = 2$, the scale parameter of the tail probability is $c = 1$ for both approximations, so the two lines are overlaid. The Stable approximation is extremely close to the simulated null distributions of $\bar{R}$ above the 95th percentile. For $\nu = 1$, the c_1 approximation is conservative in the sense of always underestimating the significance of $\bar{R}$ and the c_L approximation is anti-conservative in the sense of always overestimating the significance of $\bar{R}$ in the upper tail. For $\nu = 5$, the pattern is reversed. This behaviour indicates that the c_1 and c_L approximations do indeed represent the two endpoints for the choice of the tail scaling constant c, as intended, and that the c_1 approximation should be used when $\nu < 2$ and the c_L approximation should be used when $\nu > 2$ to ensure the test errs on the side of being conservative.

B. Robustness of the approximation to unequal prior model weights. The approximation of Equation 5 shows that the convergence of the upper tail of the null distribution of $\bar{R}$, the mean of a large number of regularly varying random variables with heavy-tail index $\lambda = 1$, is robust to unequal weights, and this property, Property 3, is shared with generalized central limit theorem (4). To demonstrate this empirically, I simulated unequal model weights independently for each simulation from a symmetric Dirichlet distribution with parameter $\mathbf{1}_L$, and used these weights

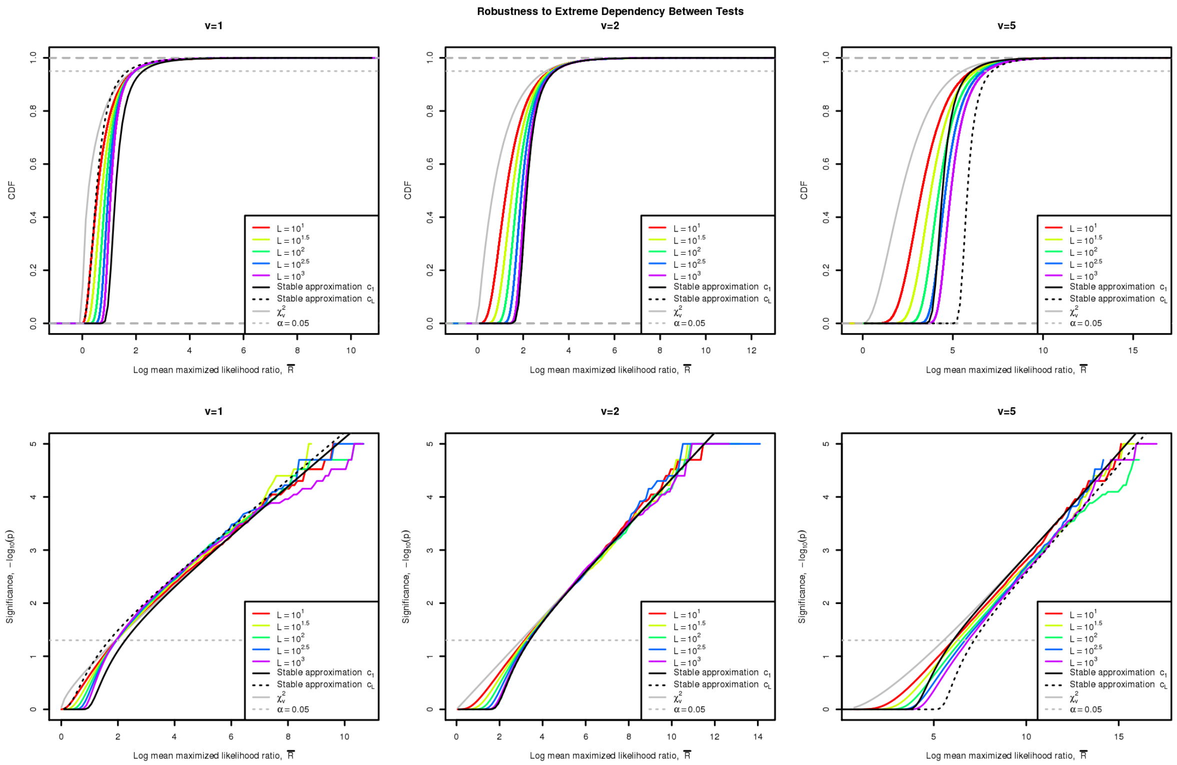

Fig. S4. Robustness of the Stable approximation to extreme dependency between tests. The simulations were conducted as for Figure S2 except that I used a resampling strategy to produce large numbers of identical tests with each simulation, producing an extreme form of dependency, i.e. identity. This involved simulating weights from a symmetric multinomial distribution, so that on average a proportion $\sim \mathrm{e}^{-1} = 0.368$ of tests were replaced by others.

to calculate $\bar{R}$ using Equation 1. Figure S3 shows that the null distributions of $\bar{R}$ with unequal model weights are extremely similar to the results with equal model weights (Figure S2), particularly in the upper tails of the distribution (lower panels) but also in the lower tails (upper panels), and even with a small number of tests $L = 10^1$ (red lines). The Stable distribution approximations performed equally well as for unequal model probabilities, particularly for $\nu = 2$, with the c_1 approximation conservative for $\nu < 2$ and the c_L approximation conservative for $\nu > 2$, as before.

C. Robustness of the approximation to extreme dependency between tests. The robustness of the Stable distribution approximation to unequal weights implies a degree of robustness to positive dependency between tests, Property 4. This is because upweighting some tests is equivalent to observing identical test statistics multiple times. Convergence of various specific forms of dependency to Stable distributions have been investigated explicitly (13), suggesting that the assumption of independence between tests might be relaxed to one of exchangeability. I simulated a form of extreme dependency between tests by simulating weights from a symmetric multinomial distribution, independently for each simulation. This is equivalent to duplicating, one or more times, some tests and completely removing others. Figure S4 shows again that the null distribution of $\bar{R}$ is very similar in the presence of this positive dependency between tests to the distribution under independence and equal model weights (Figure S2).

D. Robustness of the approximation to unequal degrees of freedom among tests. When the individual test statistics, the R_is, differ in their degrees of freedom, those with the largest degrees of freedom are expected to dominate the null distribution of $\bar{R}$ (Property 5) because their tails are the heaviest. This suggests that when, in practice, the degrees of freedom are unequal between tests, the Stable distribution approximation should be calculated using the maximum value of ν. To investigate the performance of this strategy, I simulated test statistics with two different degrees of freedom, $\nu = 1$ and $\nu = 5$ in varying proportions of 1:9, 1:1 and 9:1. Figure S5 shows that use of the c_L Stable approximation is always conservative in estimating upper tail probabilities when there is variation in the degrees of freedom, but when the proportion of tests with the maximum degrees of freedom is small, it can be substantially over-conservative. For example, when mixing tests with $\nu = 1$ and $\nu = 5$ in proportion 9:1, the c_L Stable distribution approximation under-estimated significance by an order of magnitude. As expected, the simulated distribution of $\bar{R}$ and the c_L approximation were still very similar in the upper tail, indicating that the disparity is caused by a discrepancy in the lower tail of the distribution. For such practical cases, it would be worthwhile

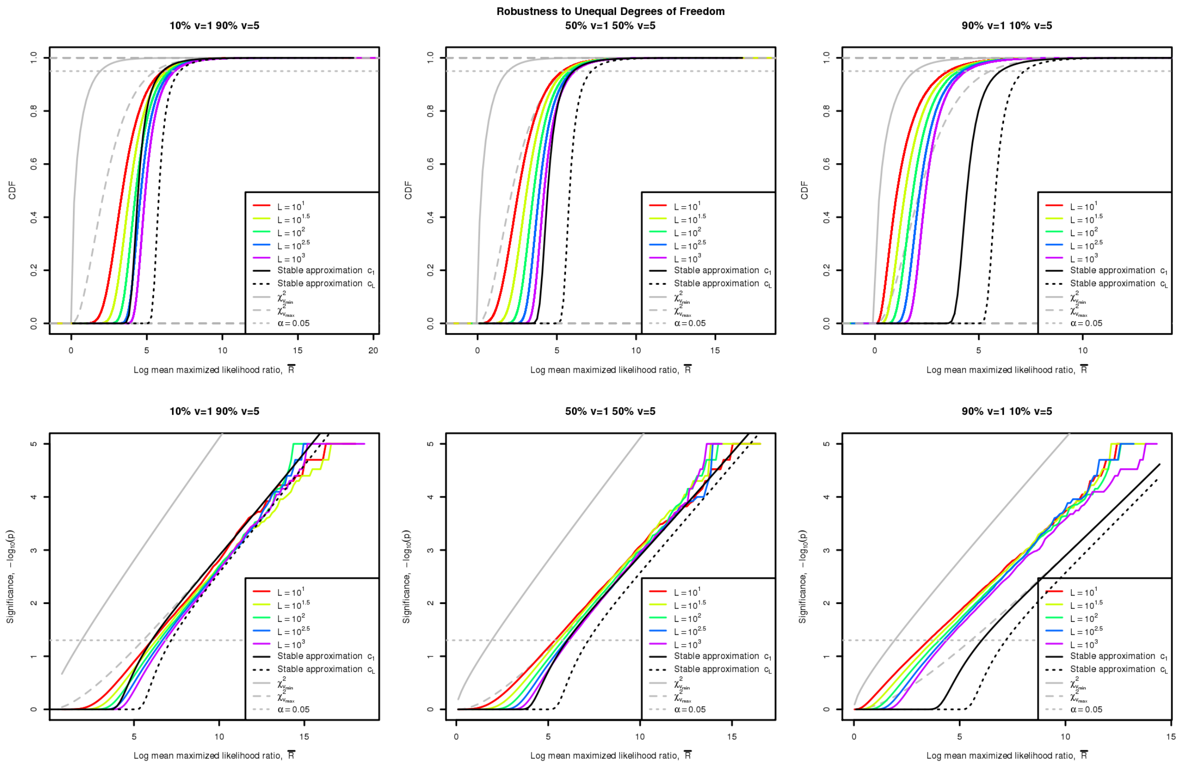

Fig. S5. Robustness of the Stable approximation to unequal degrees of freedom among tests. The simulations were conducted as for Figure S2 except in every simulation I simulated a mixture of tests with $\nu = 1$ or $\nu = 5$ degrees of freedom in three different mixture proportions: 1:9, 1:1 and 9:1.

approximating the lower tail of the null distribution of $\bar{R}$ by simulation so that

$$\Pr(\bar{r} > \bar{R}) \approx \begin{cases} 1 - F_{\text{sim}}(\bar{R}) & \text{if } \bar{R} \leq F_{\text{sim}}^{-1}(p_{\text{switch}}) \\ p_{\text{switch}} \dfrac{1 - F_{\text{Stable}}(\bar{R})}{1 - F_{\text{Stable}}(F_{\text{sim}}^{-1}(p_{\text{switch}}))} & \text{if } \bar{R} > F_{\text{sim}}^{-1}(p_{\text{switch}}) \end{cases} \qquad [30]$$

where $F_{\text{sim}}(x)$ is the empirical cumulative distribution function of a large number of simulations and the approximation switches to the Stable distribution at a large percentile such as the 99th, so that $p_{\text{switch}} = 0.99$.

E. Performance of the harmonic mean p-value. The simulations above show that the Stable distribution approximation to the null distribution of $\bar{R}$ is superior for $\nu = 2$, whereas for $\nu \neq 2$, the Stable distribution approximation is less insensitive to the number of tests L, and therefore conservative tests are required to avoid over-estimating the significance of the observed $\bar{R}$. These observations motivate the use of the HMP, $\mathring{p}$, instead of $\bar{R}$, particularly when there may be variability in the degrees of freedom between tests. Interpreting the HMP is equivalent to $\bar{R}$ when $\nu = 2$ and the assumptions of Wilks' theorem are met. When $\nu \neq 2$, interpreting the HMP is not exactly equivalent to $\bar{R}$, but it is expected to capture similar information. Partly, this is because the conversion between a maximized likelihood ratio statistic R_i and a p-value p_i is approximately locally linear for large R_i, i.e. small p_i. By Karamata's theorem, Equation 16 shows that

$$p_i \approx \frac{\log(R_i)^{\nu/2-1}}{\Gamma(\nu/2)} R_i^{-1}, \qquad R_i \to \infty. \qquad [31]$$

which is a regularly varying function, meaning that $\log(R_i)^{\nu/2-1}$ varies only slowly with respect to R_i and is approximately locally constant. Therefore the conversion from R_i to p_i is approximately linear even for $\nu \neq 2$. Moreover, the null distribution for the HMP is more robust to departures from the assumptions of Wilks' theorem, differences in degrees of freedom among tests, and the total number of tests, these latter two properties demonstrated by Figure S6. As a type of mean, it has a more intuitive form for combining tests. In section §4 I show how it also parallels the construction of the test statistic in Fisher's method for combining p-values. For these reasons, the focus of the applications of these results is on the HMP.

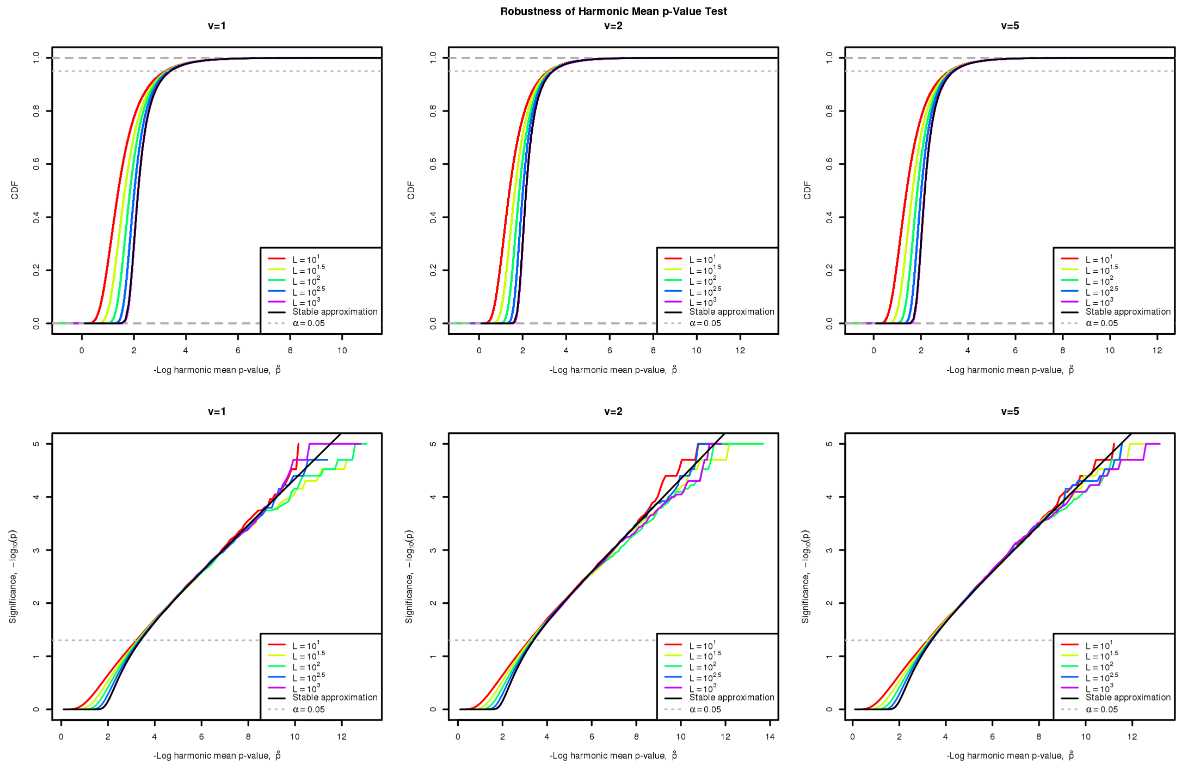

Fig. S6. Robustness of the harmonic mean p-value. The simulations were conducted as for Figure S2 except in every simulation I calculated the harmonic mean p-value instead of the mean maximized likelihood ratio $\bar{R}$. For each test I calculated a p-value p_i from the maximized likelihood ratio statistic R_i with $\nu = 1$, 2 or 5 degrees of freedom, and from them I calculated the harmonic mean. The distribution of the HMP is insensitive to the degrees of freedom, requiring only that the individual p-values are correctly calibrated.

3. Controlling the strong-sense family-wise error rate of the HMP test

Limiting the probability with which a combined test falsely rejects the null hypotheses when it is true is known as weak-sense control of the family-wide error rate (FWER). Strong-sense control of the FWER additionally entails limiting the probability with which any of the true null hypotheses is rejected when one or more null hypotheses are false. A procedure that controls the strong-sense FWER can be constructed from any method that controls the weak-sense FWER using the *closure principle* (14). Consider a situation in which a series of not necessarily independent binary hypothesis tests are conducted, each one between a null hypothesis $\mathcal{O}$ and an alternative $\mathcal{A}$ and define

$$\begin{array}{rcl} U_i & : & \mathcal{O}_i \text{ is preferred to } \mathcal{A}_i \\ U_i \cap U_j & : & \mathcal{O}_i \text{ is preferred to } \mathcal{A}_i \textbf{ and } \mathcal{O}_j \text{ is preferred to } \mathcal{A}_j \end{array} \qquad [32]$$

and by implication

$$\begin{array}{rcl} U_i' & : & \mathcal{A}_i \text{ is preferred to } \mathcal{O}_i \\ (U_i \cap U_j)' & : & \mathcal{A}_i \text{ is preferred to } \mathcal{O}_i \text{ and } \mathcal{A}_j \text{ is preferred to } \mathcal{O}_j \textbf{ or} \\ & & \mathcal{A}_i \text{ is preferred to } \mathcal{O}_i \text{ and } \mathcal{O}_j \text{ is preferred to } \mathcal{A}_j \textbf{ or} \\ & & \mathcal{O}_i \text{ is preferred to } \mathcal{A}_i \text{ and } \mathcal{A}_j \text{ is preferred to } \mathcal{O}_j. \end{array} \qquad [33]$$

The null hypothesis associated with an individual U_i is called an *elementary* hypothesis while I will call intersections of null hypotheses associated with $\{U_i \cap U_j\}$ *compound* hypotheses. Each test is associated with a p-value assumed to follow a Uniform$(0, 1)$ distribution if the elementary or compound hypothesis is true. The *closure* of the elementary hypotheses is the set of all possible intersections involving one or more elementary hypotheses. For example, for the elementary hypotheses $\{U_1, U_2, U_3\}$, the closure is

$$\{\{U_1\}, \{U_2\}, \{U_3\}, \{U_1 \cap U_2\}, \{U_1 \cap U_3\}, \{U_2 \cap U_3\}, \{U_1 \cap U_2 \cap U_3\}\}\,.$$

In a *closed testing procedure* (14) (CTP), an elementary hypothesis U_i is only rejected if *every intersection hypothesis involving* U_i *is also rejected*. For example, U_1 is only rejected if $\{U_1 \cap U_2\}$, $\{U_1 \cap U_3\}$ and $\{U_1 \cap U_2 \cap U_3\}$ are also rejected.

Suppose $\mathcal{R} \subseteq \{1, 2, \ldots, L\}$ is a set indexing some or all of the L elementary hypotheses $U_1 \ldots U_L$, $\psi_{\mathcal{R}}$ is a test that controls the weak-sense FWER at level α for the compound hypothesis $U_{\mathcal{R}} = \{\cap_{i \in \mathcal{R}} U_i\}$ and $p_{\psi_{\mathcal{R}}}$ is the corresponding p-value. Then the following test controls the strong-sense FWER at level α:

$$\phi_{\mathcal{R}} = \begin{cases} 1 & \text{if } \max\{p_{\psi_{\mathcal{Q}}} : \mathcal{Q} \supseteq \mathcal{R}\} \leq \alpha & \text{meaning } U_{\mathcal{R}} \text{ is rejected} \\ 0 & \text{otherwise} & \text{meaning } U_{\mathcal{R}} \text{ is not rejected} \end{cases} \qquad [34]$$

A. Derivation of the closed testing procedure. Suppose $\mathcal{R}_0$ is the **unknown** set indexing only the correct elementary hypotheses: this implies the existence of $\mathcal{R}_0'$, a (possibly empty) complementary set indexing the incorrect elementary hypotheses. Define the following events:

A : One or more of the correct elementary hypotheses $U_i, i \in \mathcal{R}_0$ is rejected by the CTP

B : The compound hypothesis comprising the intersection of correct elementary hypotheses $\bigcap_{i \in \mathcal{R}_0} U_i$ is rejected

Note that

- Since the CTP uses a method that controls the weak-sense FWER at each step, $\Pr(B) \leq \alpha$
- $A \subseteq B$ because the CTP rejects subset hypotheses only after it has rejected all their superset hypotheses, so $\Pr(A) = \Pr(A \cap B) = \Pr(B)\Pr(A \mid B) \leq \alpha$
- It doesn't matter that $\mathcal{R}_0$ is unknown because these properties are guaranteed for any possible set of true null hypotheses.

This is the standard definition for the strong-sense FWER, and it is robust to non-independence between tests within the set of true null hypotheses ($\mathcal{R}_0$). However, its first assumption could be violated by correlation between the p-values of the true null hypotheses and true alternative hypotheses. If this is invalid then control of the weak-sense FWER, and therefore the strong-sense FWER, cannot be guaranteed. This caveat would seem to apply to any CTP.

B. Bonferroni achieves strong-sense FWER. Suppose that $\psi_{\mathcal{R}}$ records the result of a single weighted Bonferroni test controlling the weak-sense FWER for whether the intersection of elementary hypotheses indexed by set $\mathcal{R}$, $U_{\mathcal{R}} = \{\cap_{i \in \mathcal{R}} U_i\}$, is rejected:

$$\psi_{\mathcal{R}} = \begin{cases} 1 & \text{if } \min\left\{\dfrac{p_i\, w_{\mathcal{R}}}{w_i} : i \in \mathcal{R}\right\} \leq \alpha & \text{meaning } U_{\mathcal{R}} \text{ is rejected} \\ 0 & \text{otherwise} & \text{meaning } U_{\mathcal{R}} \text{ is not rejected} \end{cases} \qquad [35]$$

where $w_{\mathcal{R}} = \sum_{i \in \mathcal{R}}^{L} w_i$. A naive implementation of CTP involves $(2^L - 1)$ of these tests. But in practically useful implementations of CTP, shortcuts are found which mean not all tests need performing. In the case of Bonferroni, the test

$$\phi_{R} = \begin{cases} 1 & \text{if } \min\left\{\dfrac{p_i\, w_{\mathcal{R}}}{w_i} : i \in \mathcal{R}\right\} \leq \alpha\, w_{\mathcal{R}} & \text{meaning } U_{\mathcal{R}} \text{ is rejected} \\ 0 & \text{otherwise} & \text{meaning } U_{\mathcal{R}} \text{ is not rejected} \end{cases} \qquad [36]$$

is a shortcut procedure that satisfies the CTP because its rejection implies all intersection hypotheses involving the elementary hypotheses indexed by $\mathcal{R}$ will also be rejected, a property that is easier to see by rewriting the test, cancelling the $w_{\mathcal{R}}$ term on both sides. This means that only the L elementary hypotheses need directly testing to control the strong-sense FWER at level α using the Bonferroni test.

C. A multilevel CTP for the HMP. By the same argument, testing significance by directly interpreting the HMP as per Equation 6,

$$\phi_{R} = \begin{cases} 1 & \text{if } \overset{\circ}{p}_R \leq \alpha\, w_R & \text{meaning } R \text{ is rejected} \\ 0 & \text{otherwise} & \text{meaning } R \text{ is not rejected} \end{cases}$$

is a multilevel CTP controlling the strong-sense FWER at level approximately α, as shown by Equation 7. By this test, any set that contains a significant subset is itself significant and, conversely, if the set of all hypotheses is not significant, no subset is significant. However, this shortcut procedure is only approximate because the exact

significance threshold varies by the number of hypotheses combined (Table 1). The full CTP that controls the strong-sense FWER without sacrificing power is derived from Equation 34 as

$$\phi_{\mathcal{R}} = \begin{cases} 1 & \text{if } \max\left\{\frac{\mathring{p}_{\mathcal{Q}}}{\alpha_{|\mathcal{Q}|}} : \mathcal{Q} \supseteq \mathcal{R}\right\} \leq 1 & \text{meaning } U_{\mathcal{R}} \text{ is rejected} \\ 0 & \text{otherwise} & \text{meaning } U_{\mathcal{R}} \text{ is not rejected} \end{cases} \quad [37]$$

The disadvantage of this CTP is that many p-values must be considered for each test. Shortcut procedures can be defined that improve on a naive implementation of Equation 37 but instead one can apply weighted Bonferroni correction to make a simple adjustment to Equation 6 by substituting $\alpha_{|\mathcal{R}|}$ for α. (Equivalently, one can apply weighted Bonferroni correction to Equation 4.) This produces a more conservative multilevel CTP than Equation 37 that is nevertheless guaranteed to control the strong-sense FWER, up to the order of the Stable distribution approximation.

In practice, control of the strong-sense FWER can be achieved by a two-pass procedure in which the more lenient threshold α is used initially to discover candidate significant hypotheses, which are then confirmed using the exact threshold $\alpha_{|\mathcal{R}|}$.

4. The relationship between the HMP and other combined tests

A. The HMP is closely related to but more powerful than Simes' procedure. In this section I show that the HMP is closely related to Simes' test (15). This provides a new, intuitive interpretation of the Simes test as an approximation to the harmonic mean p-value by showing that it provides the smallest upper bound on the HMP based on a single p-value. Simes' test, as extended by Hochberg and Liberman (16) to include unequal weights, can be written as

$$\psi_{\mathcal{R}} = \begin{cases} 1 & \text{if } \min\left\{\frac{p^*_{(i)}\, w_{\mathcal{R}}}{i} : i = 1 \ldots |\mathcal{R}|, p^*_j = \frac{p_j}{w_j}, j \in \mathcal{R}\right\} \leq \alpha & \text{meaning } U_{\mathcal{R}} \text{ is rejected} \\ 0 & \text{otherwise} & \text{meaning } U_{\mathcal{R}} \text{ is not rejected} \end{cases} \quad [38]$$

where $p^*_{(i)}$ is the ith smallest value of the p^*_js and $w_{\mathcal{R}} = \sum_{i\in\mathcal{R}} w_i$. This test controls the weak-sense FWER at level α when $\max_i w_i/w_{\mathcal{R}} \leq 1/(\alpha\,|\mathcal{R}|)$, and is conservative otherwise. The following modified test defines a shortcut multilevel CTP that controls the strong-sense FWER:

$$\phi_{\mathcal{R}} = \begin{cases} 1 & \text{if } \min\left\{\frac{p^*_{(i)}\, w_{\mathcal{R}}}{i} : i = 1 \ldots |\mathcal{R}|, p^*_j = \frac{p_j}{w_j}, j \in \mathcal{R}\right\} \leq \alpha\, w_{\mathcal{R}} & \text{meaning } U_{\mathcal{R}} \text{ is rejected} \\ 0 & \text{otherwise} & \text{meaning } U_{\mathcal{R}} \text{ is not rejected} \end{cases} \quad [39]$$

where $\sum_{i=1}^{L} w_i = 1$. Like the Bonferroni and HMP shortcut procedures, a set of alternative hypotheses will be significant by this modified Simes' test if any subset of those alternative hypotheses is significant. This is because when the rank of the p^*_j defining the minimum test statistic in set $\mathcal{R}$ is i, its rank in any superset must be at least i as well. However, the converse is not certain.

To facilitate the comparison between Simes' test statistic and the HMP, it can be rewritten in terms of 'b-values' (inverse p-values) as

$$b_{\text{Simes}} = \max\left\{\frac{b^*_{[i]}\, i}{w_{\mathcal{R}}} : i = 1 \ldots |\mathcal{R}|, b^*_j = w_j\, b_j, j \in \mathcal{R}\right\} \quad [40]$$

where $b^*_{[i]}$ denotes the ith largest value of the b^*_js. Compare this to the inverse HMP,

$$\bar{b}_{\mathcal{R}} = \frac{\sum_{i\in\mathcal{R}} w_i\, b_i}{w_{\mathcal{R}}}. \quad [41]$$

When b_{Simes} is maximized at the largest, second largest and third largest of the b^*_js respectively, it is clear that

$$\begin{aligned} & & & \frac{b^*_{[1]}}{w_{\mathcal{R}}} & & \leq \bar{b} \\ \frac{b^*_{[1]}}{w_{\mathcal{R}}} & \leq & \frac{2\,b^*_{[2]}}{w_{\mathcal{R}}} & \leq & \frac{b^*_{[1]} + b^*_{[2]}}{w_{\mathcal{R}}} & \leq \bar{b} \\ \max\left\{\frac{b^*_{[1]}}{w_{\mathcal{R}}}, \frac{2\,b^*_{[2]}}{w_{\mathcal{R}}}\right\} & \leq & \frac{3\,b^*_{[3]}}{w_{\mathcal{R}}} & \leq & \frac{b^*_{[1]} + b^*_{[2]} + b^*_{[3]}}{w_{\mathcal{R}}} & \leq \bar{b} \end{aligned}$$

By extension, the Simes test statistic $p_{\text{Simes}} = 1/b_{\text{Simes}}$ approximates the harmonic mean p-value $\overset{\circ}{p}_{\mathcal{R}} = 1/\bar{b}_{\mathcal{R}}$, providing the smallest upper bound on the HMP possible using a single p-value. This means the behaviour of the two tests should be very similar. Indeed, not only has Simes' procedure been extended to unequal weights (16), it has also been shown to be robust to positive dependence between tests (17). Like the HMP, it combines tests adaptively, producing a Bonferroni-like p-value for 'needle-in-a-haystack' problems when one test dominates, but capitalizing on numerous strongly significant or suggestive tests when warranted to produce a smaller combined p-value. However, it makes less use of the data and does not share the property of the HMP that when the test is not significant, neither is any subset of the constituent tests, suggesting it may be less statistically efficient, and therefore less powerful. To investigate this, I conducted power simulations comparing Bonferroni, Simes and HMP tests.

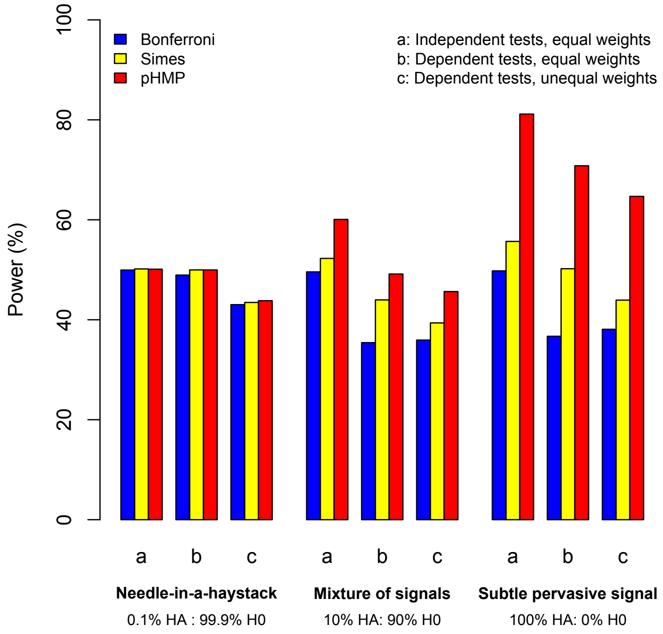

Fig. S7. Comparison of the power of the Bonferroni, Simes' and HMP ($\overset{\circ}{p}$, Equation 4) tests across a range of simulated scenarios. In each scenario, 10 000 simulations of $L = 1000$ tests were performed and the proportion of times the combined p-value was below the significance threshold of $\alpha = 0.05$ was recorded. Each simulation comprised a mixture of tests drawn from the null hypothesis 'H0' ($p_i \sim \text{Uniform}(0, 1)$) and the alternative hypothesis 'HA' ($p_i \sim \text{Beta}(1/\theta, 1)$) in varying proportions indicated. The parameter $\theta > 1$ was chosen to standardize the power of the Bonferroni test to 50% when tests were simulated independently with equal weights (setting a). This generated a smaller θ and subtler distinction between null and alternative hypotheses when a larger proportion of the tests were drawn from the alternative hypothesis. In setting b, dependency was simulated by resampling tests uniformly at random. Setting c additionally simulated poorly chosen weights, which were drawn from a Beta$(1/2, 1)$ distribution and normalized to sum to one.

Figure S7 shows that except for the 'Needle-in-a-haystack' scenario, in which the alternative hypothesis is true for only a single test, the HMP substantially outperformed both Bonferroni and Simes' tests. Under a 'Mixture of signals' scenario, in which the alternative hypothesis was true for 10% of tests, the HMP outperformed Simes and Bonferroni by 14% and 29% respectively. Under a 'Subtle pervasive signal' scenario, in which the alternative hypothesis was true for all tests, but differed only subtly from the null hypothesis, the HMP outperformed Simes and Bonferroni by 45% and 75% respectively. Even under the needle-in-a-haystack scenario, where Simes' and the HMP test performed similarly, they slightly outperformed Bonferroni correction by 1.2% and 1.4% respectively. The power of all tests was adversely affected by dependency and poorly-chosen, unequal weights, but the relative performance characteristics of the tests remained similar.

B. Complementarity to Fisher's method for combining p-values. Fisher's method (18, p. 103) provides another procedure for combining p-values that is often very powerful, but makes a strong assumption of independence between the tests. As I will show, Fisher's method and the HMP can be seen as complementary methods for combining tests when the alternative hypotheses are independent or mutually exclusive respectively. The test statistic for Fisher's method can be written as the sum of the 'significances', expressed as the $-\log_e p$-values, of the individual tests:

$$\sum_{i \in \mathcal{R}} -\log p_i.$$

Under the null hypothesis, this quantity follows a Gamma$(\alpha = |\mathcal{R}|, \beta = 1)$ distribution (18). Fisher's method can be expressed as

$$\psi_{\mathcal{R}} = \begin{cases} 1 & \text{if } 1 - F_{\text{Gamma}}\left(\sum_{i \in \mathcal{R}} -\log p_i \,|\, \alpha = |\mathcal{R}|, \beta = 1\right) \leq \alpha & \text{meaning } U_{\mathcal{R}} \text{ is rejected} \\ 0 & \text{otherwise} & \text{meaning } U_{\mathcal{R}} \text{ is not rejected} \end{cases} \quad [42]$$

As for the HMP, Fisher's method can be interpreted in terms of a likelihood ratio test, an interpretation that is exact when the individual p-values are derived from likelihood ratio tests with $\nu = 2$ degrees of freedom. In this form, it

becomes apparent that Fisher's method combines a series of tests by testing the grand alternative hypothesis against the grand null:

$$\begin{aligned}\exp\left\{\sum_{i\in\mathcal{R}} -\log p_i\right\} &= \prod_{i\in\mathcal{R}} R_i = \prod_{i\in\mathcal{R}} \frac{\Pr\left(\mathbf{X}_i|\mathcal{A}_i\right)}{\Pr\left(\mathbf{X}_i|\mathcal{O}_i\right)} \\ &= \frac{\Pr\left(\{\mathbf{X}_i : i\in\mathcal{R}\}\mid \cap_{i\in\mathcal{R}}\mathcal{A}_i\right)}{\Pr\left(\{\mathbf{X}_i : i\in\mathcal{R}\}\mid \cap_{i\in\mathcal{R}}\mathcal{O}_i\right)},\end{aligned} \qquad [43]$$

where $\mathcal{O}_i$ and $\mathcal{A}_i$ represent the nested null and alternative hypothesis associated with likelihood ratio test i.

In contrast, the HMP can be interpreted in the same circumstances as testing a model-averaged alternative hypothesis against the grand null in which each individual alternative hypothesis is treated as mutually exclusive from the others:

$$\begin{aligned}\overset{\circ}{p}_{\mathcal{R}}^{-1} = \frac{\sum_{i\in\mathcal{R}} w_i/p_i}{\sum_{i\in\mathcal{R}} w_i} &= \frac{\sum_{i\in\mathcal{R}} w_i\, R_i}{\sum_{i\in\mathcal{R}} w_i} = \sum_{i\in\mathcal{R}} \frac{w_i}{w_{\mathcal{R}}}\frac{\Pr\left(\mathbf{X}_i|\mathcal{A}_i\right)}{\Pr\left(\mathbf{X}_i|\mathcal{O}_i\right)} \\ &= \frac{\sum_{i\in\mathcal{R}} \Pr\left(A_i \cap_{j\in\mathcal{R}\setminus i} O_j\right)\, \Pr\left(\mathbf{X}_i|\mathcal{A}_i\right)\prod_{j\in\mathcal{R}\setminus i}\Pr\left(\mathbf{X}_j|\mathcal{O}_j\right)}{\prod_{i\in\mathcal{R}}\Pr\left(\mathbf{X}_i|\mathcal{O}_i\right)} \\ &= \frac{\Pr\left(\{\mathbf{X}_i : i\in\mathcal{R}\}\mid \cup_{i\in\mathcal{R}}\mathcal{A}_i\cap_{j\in\mathcal{R}\setminus i}\mathcal{O}_j\right)}{\Pr\left(\{\mathbf{X}_i : i\in\mathcal{R}\}\mid \cap_{i\in\mathcal{R}}\mathcal{O}_i\right)}.\end{aligned} \qquad [44]$$

Here the model weights, the w_is, are interpreted as proportional to prior model probabilities on the mutually exclusive alternative hypotheses. A more detailed consideration of the role of the weights is provided in section §5C.

Thus, the interpretation of what it means to reject a compound hypothesis (Equation 33) depends on the test. For example, if U_i represents the acceptance of the null $\mathcal{O}_i$ over the alternative $\mathcal{A}_i$, and $(U_i \cap U_j)'$ represents the rejection of the compound hypothesis $U_i \cap U_j$, then in the case of Fisher's method this corresponds most closely to

$$(U_i \cap U_j)' \quad : \quad \mathcal{A}_i \text{ is preferred to } \mathcal{O}_i \text{ and } \mathcal{A}_j \text{ is preferred to } \mathcal{O}_j$$

whereas in the case of the HMP it corresponds most closely to

$$\begin{aligned}(U_i \cap U_j)' \quad : \quad & \mathcal{A}_i \text{ is preferred to } \mathcal{O}_i \text{ and } \mathcal{O}_j \text{ is preferred to } \mathcal{A}_j \textbf{ or} \\ & \mathcal{O}_i \text{ is preferred to } \mathcal{A}_i \text{ and } \mathcal{A}_j \text{ is preferred to } \mathcal{O}_j.\end{aligned}$$

While the data *must* be independent for Fisher's method to be valid, in the case of the HMP, the data do not need to be independent. In fact the same data can appear in multiple tests, in which case terms in Equation 44 will cancel, so that in the case where the same data and same null hypothesis are used for every test, the interpretation simplifies to

$$\overset{\circ}{p}_{\mathcal{R}}^{-1} = \frac{\sum_{i\in\mathcal{R}}\Pr\left(A_i\right)\,\Pr\left(\mathbf{X}|\mathcal{A}_i\right)}{\Pr\left(\mathbf{X}|\mathcal{O}\right)}, \qquad [45]$$

as originally intended in Equation 1. This flexibility formalizes the idea that the HMP can be applied not only to p-values constructed from a series of likelihood ratio tests for the same data, same nested null hypothesis and different alternative hypotheses, but can be applied more widely as a general-purpose method for combining p-values. Indeed, there may be cases when imposing the constraint that the alternative hypotheses are mutually exclusive is more powerful than testing the grand alternative against the grand null, as in Fisher's method. In this respect, the HMP is a complementary tool to Fisher's method.

To investigate the power of the HMP relative to Fisher's method, and to illustrate situations in which the HMP is appropriate but Fisher's method is not, I repeated the simulation study from the previous subsection, including Fisher's method for combining p-values. For needle-in-a-haystack problems, Fisher's method showed considerably less power than Bonferroni, Simes' and the HMP tests (Figure S8). This can be understood through the interpretation of Fisher's test as comparing the grand alternative to the grand null. When only 0.1% of tests are drawn from the alternative hypothesis, the distribution of p-values resembles the grand null more closely than the grand alternative, resulting in a 66% reduction in power compared to the HMP.

In the two other scenarios, where there were a mixture of signals or a subtle pervasive signal, the power of Fisher's method was 60% and 40% greater than the HMP respectively. However, in the presence of non-independence between tests, the superior performance of Fisher's test was counterbalanced by an extremely high false positive rate of 12%, around 2.5 times higher than the specified false positive rate of $\alpha = 5\%$. This reveals a trade-off between power and robustness: the assumption of independence between tests contributes to considerably higher power for Fisher's method, but the test is not robust when that assumption is violated, leading to inadmissibly high false positive rates.

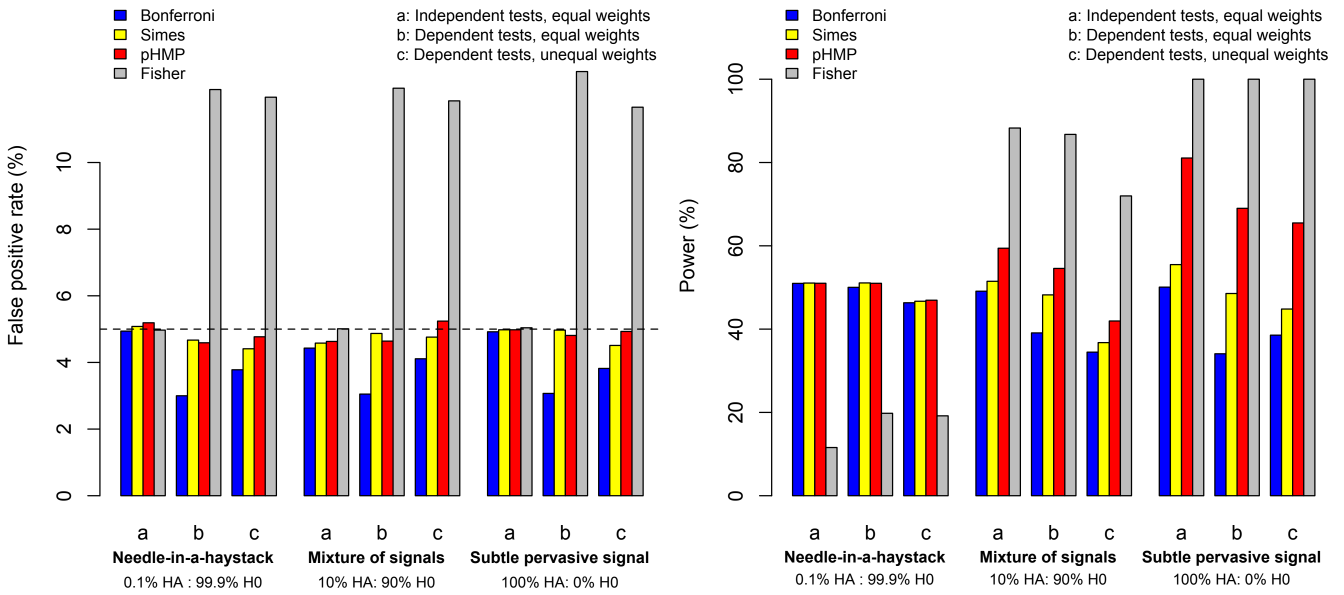

Fig. S8. Comparison of the false positive rate and power of the Bonferroni, Simes', HMP ($\overset{\circ}{p}$, Equation 4) and Fisher's tests across a range of simulated scenarios. Simulations were conducted as per Figure S7, for both the false positive rate (left) and power (right). To calculate false positive rates, the parameter θ was set to one throughout; the three scenarios are therefore equivalent for this purpose. Fisher's method does not account for weights, so these were ignored.

C. Position within Loughin's classification of methods for combining p-values. Loughin (19) classified methods for combining p-values from independent tests into two broad categories. In the first, each p-value is transformed via an inverse cumulative distribution function into a variable that is then summed to produce the combined test statistic,

$$\begin{aligned} Y_{\mathcal{R}} &= \sum_{i\in R} F^{-1}(1-p_i), \\ \text{so that} \quad \psi_{\mathcal{R}} &= \begin{cases} 1 & \text{if } \Pr\left(y_{\mathcal{R}} \geq Y_{\mathcal{R}}\right) \leq \alpha \quad \text{meaning } U_{\mathcal{R}} \text{ is rejected} \\ 0 & \text{otherwise} \quad \text{meaning } U_{\mathcal{R}} \text{ is not rejected} \end{cases} \end{aligned} \qquad [46]$$

Loughin provided examples including Fisher's method that utilizes the chi-squared distribution with $\nu = 2$ degrees of freedom (18), Lancaster's that allows variable degrees of freedom (20), Lipták's that utilises the standard normal distribution (21), Edgington's that utilizes the uniform distribution (22) and Mudholkar and George's that utilizes the logistic function (23). The second broad category of methods define the test statistic in terms of an order statistic of the observed p-values. Again, this is compared against a null distribution to determine whether it is significantly extreme. Examples include the minimum and maximum p-values. With respect to this classification, the HMP appears to represent a novel method belonging to the first broad category, in which the transforming inverse cumulative distribution function is the Pareto distribution, with $F^{-1}(1-p_i) = 1/p_i$.

5. Bayesian connections and comparing competing alternative hypotheses

A. Similarity between Bayesian model-averaged significance testing and the HMP. It is widely recounted that Bonferroni correction for multiple testing is conservative, especially when the tests are not independent, but comparison to Bayesian procedures suggests this view may be over-stated in a specific sense. As a means of *combining* tests, section §4 shows that the HMP and Simes' test comfortably outperform Bonferroni, except for 'needle-in-a-haystack' problems. However, as a means of testing whether a *specific* individual alternative hypothesis is significantly better than the null hypothesis, taking into account the total number of alternative hypotheses, the Bonferroni procedure is appropriate. The Bonferroni procedure utilizes Boole's inequality to control the family-wise error rate (FWER) at level α, or some more stringent level below α:

$$\Pr\left(\min_{i\in\mathcal{R}}\left\{p_i\frac{w_{\mathcal{R}}}{w_i}\right\}\leq\alpha\right) = \Pr\left(\bigcup_{i\in\mathcal{R}} p_i\frac{w_{\mathcal{R}}}{w_i}\leq\alpha\right) \leq \sum_{i\in\mathcal{R}}\Pr\left(p_i\frac{w_{\mathcal{R}}}{w_i}\leq\alpha\right) = \sum_{i\in\mathcal{R}}\alpha\frac{w_i}{w_{\mathcal{R}}} = \alpha. \qquad [47]$$

The FWER is controlled at exactly level α if only one p-value can be significant at once, which is not usually the case. While this explains why *combined* tests have been devised that control the FWER at level *exactly* α, and are therefore more powerful, a comparison to the Bayesian approach suggests that the Bonferroni procedure is not a fundamentally conservative approach to assessing the significance of a *specific* alternative hypothesis, correcting for the multiple alternative hypotheses.

This issue is relevant to the HMP short-cut procedure (Equation 6) which improves power by simultaneously performing combined tests at multiple levels, but for which the rejection of the null hypothesis in favour of a specific alternative hypothesis, or set of alternative hypotheses, $\mathcal{R}$, is subject to the combined p-value, $\overset{\circ}{p}_{\mathcal{R}}$, falling below the Bonferroni threshold $\alpha\, w_{\mathcal{R}}$.

For the purpose of comparison to the Bayesian approach, suppose that the alternative hypotheses are mutually exclusive. This assumption is not restrictive because the outcomes of any group of non-mutually exclusive tests can, in principle, be fully enumerated to form an exhaustive list of mutually exclusive outcomes. Consider L mutually exclusive alternative hypotheses $\mathcal{M}_i$, $i = 1 \dots L$ and a common null hypothesis $\mathcal{M}_0$. The HMP employs weights w_i, $i = 1 \dots L$ where $\sum_{i=1}^{L} w_i = 1$, while the Bayesian procedure employs prior probabilities for the null hypothesis (μ_0) and each alternative $(1-\mu_0)\,\mu_i$, where $\sum_{i=1}^{L} \mu_i = 1$. Denote the Bayes factor comparing $\mathcal{M}_i$ to $\mathcal{M}_0$ as BF_i and, for the purposes of comparison, define a 'b-value' to be an inverse p-value, so that $b_i = p_i^{-1}$.

In the Bayesian model comparison setting, the null hypothesis is rejected when it does not fall within the smallest $100(1-\alpha)\%$ credible set of models. In the classical setting, the null hypothesis is rejected when the p-value falls below the relevant significance threshold. Thus, the Bayesian and HMP procedures reject the null hypothesis $\mathcal{M}_0$ in favour of a specific alternative hypothesis $\mathcal{M}_i$ when

Bayesian			HMP		
$\dfrac{\Pr(\mathcal{M}_i \mid \mathbf{X})}{\Pr(\mathcal{M}_0 \mid \mathbf{X})} = \dfrac{\Pr(\mathcal{M}_i)}{\Pr(\mathcal{M}_0)} \dfrac{\Pr(\mathbf{X} \mid \mathcal{M}_i)}{\Pr(\mathbf{X} \mid \mathcal{M}_0)}$	$\geq$	$\dfrac{1-\alpha}{\alpha}$	$\alpha\, w_i$	$\geq$	p_i
$\dfrac{\alpha\,(1-\mu_0)}{\mu_0\,(1-\alpha)}\, \mu_i\, \text{BF}_i$	$\geq$	1	$\alpha\, w_i\, b_i$	$\geq$	1

The two procedures reject the null hypothesis $\mathcal{M}_0$ in favour of the model-averaged alternative hypothesis $\mathcal{M}_{\mathcal{R}}$ when

Bayesian			HMP		
$\dfrac{\Pr(\mathcal{M}_{\mathcal{R}} \mid \mathbf{X})}{\Pr(\mathcal{M}_0 \mid \mathbf{X})} = \dfrac{\sum_{i \in \mathcal{R}} \Pr(\mathcal{M}_i) \Pr(\mathbf{X} \mid \mathcal{M}_i)}{\Pr(\mathcal{M}_0) \Pr(\mathbf{X} \mid \mathcal{M}_0)}$	$\geq$	$\dfrac{1-\alpha}{\alpha}$	$\alpha_{\lvert\mathcal{R}\rvert}\, w_{\mathcal{R}}$	$\geq$	$\overset{\circ}{p}_{\mathcal{R}}$
$\dfrac{\alpha\,(1-\mu_0)}{\mu_0\,(1-\alpha)} \sum_{i \in \mathcal{R}} \mu_i\, \text{BF}_i$	$\geq$	1	$\alpha_{\lvert\mathcal{R}\rvert} \sum_{i \in \mathcal{R}} w_i\, b_i$	$\geq$	1

Since, for small α (e.g. $\alpha \leq 0.05$), $\alpha_{\lvert\mathcal{R}\rvert} \approx \alpha$, this shows there exists a combination of probability threshold α, weights $\{w_i, 1 \leq i \leq L\}$, prior odds μ_0, and prior probabilities $\{\mu_i, 1 \leq i \leq L\}$ for which the Bayesian procedure would reject the null in favour of the same set of alternative hypotheses and model-averaged alternative hypotheses as the HMP. Note however that the scales of the Bayes factor and the b-value are not identical. Nevertheless, this reiterates the observation that the inverse p-values interact approximately linearly, like Bayes factors, at least when they are large. This relationship is formalized in a Bayesian interpretation of the HMP below.

The parallels between the Bayesian approach to model-averaged hypothesis testing and the HMP are instructive, because they offer an alternative motivation for the underlying Bonferroni multiple-testing correction that does not view the procedure as fundamentally flawed because it is conservative, but rather as a natural approach to testing specific hypotheses or groups of hypotheses. For advocates of the false discovery rate (FDR), for whom control of the FWER is conservative, the Bonferroni procedure is doubly conservative. Yet the Bayesian connection, which is developed further below, suggests that contrary to this widespread critique, both Bonferroni correction and, by implication, the FWER are frequentist analogs to natural Bayesian procedures.

B. Parallels between Bayesian model-averaging and Simes' method. Simes' procedure also parallels the Bayesian model-averaging approach, and this demonstrates the close connection between the HMP and Simes' procedure. The

two procedures reject the null hypothesis $\mathcal{M}_0$ in favour of the alternative set of hypotheses $\mathcal{M}_\mathcal{R}$ when

Bayesian			Simes		
$\frac{\Pr(\mathcal{M}_\mathcal{R}\|\mathbf{X})}{\Pr(\mathcal{M}_0\|\mathbf{X})} \geq \frac{\max_{1\leq i\leq\|\mathcal{R}\|}\left\{i\,\{\Pr(\mathcal{M}_j)\Pr(\mathbf{X}\|\mathcal{M}_j) : j\in\mathcal{R}\}_{[i]}\right\}}{\Pr(\mathcal{M}_0)\Pr(\mathbf{X}\|\mathcal{M}_0)}$	$\geq$	$\frac{1-\alpha}{\alpha}$	$\alpha\, w_\mathcal{R}$	$\geq$	p_{Simes}
$\frac{\alpha\,(1-\mu_0)}{\mu_0\,(1-\alpha)}\max_{1\leq i\leq\|\mathcal{R}\|}\left\{i\,\{\mu_j\,\text{BF}_j : j\in\mathcal{R}\}_{[i]}\right\}$	$\geq$	1	$\alpha\max_{1\leq i\leq\|\mathcal{R}\|}\left\{i\,\{w_j\,b_j : j\in\mathcal{R}\}_{[i]}\right\}$	$\geq$	1

This reiterates that there exists a combination of significance threshold, weights and prior model probabilities for which the Bayesian procedure would reject the null in favour of the same set of alternative hypotheses as the classical procedure, in this case Simes'. It also reiterates the lower statistical efficiency of Simes' procedure compared to the HMP because the Bayesian criterion analogous to Simes' procedure for rejecting the null hypothesis will always be smaller, and therefore less powerful, than the Bayesian criterion analogous to the HMP for the same data.

In these tests, the ith largest value of the weighted Bayes factor or weighted b-value defines the minimum threshold α at which the null hypothesis would be rejected. This means that when the test is marginal in significance, all the top i alternative hypotheses are needed as a group to reject the null, and only groups of alternative hypotheses including them will be significant.

Simes also proposed the Benjamini-Hochberg (BH) procedure as an exploratory tool for prioritizing alternative hypotheses for further investigation (15). Benjamini and Hochberg subsequently showed (24) that this procedure controls the expected false discovery rate (FDR) at level α. In the BH procedure, the null is rejected in favour of all alternative hypotheses $\mathcal{M}_i$ for which

$$\alpha\, i\,\{w_j\,b_j : j\in\mathcal{R}\}_{[i]} \geq 1.$$

This seems to define the set of alternative hypotheses that, together with any $(i-1)$ alternatives with larger weighted b-values, would reject the null hypothesis in favour of the i alternatives. In this sense, the null is really rejected in favour of the alternatives as *groups* or *sets* of increasing size, rather than individually. That the HMP makes this interpretation explicit can be seen as another advantage over the BH procedure.

C. Interpretation of the harmonic mean p-value as a Bayesian procedure. The HMP can be interpreted as directly proportional to a Bayes factor, subject to the following assumptions:

1. The distribution of the p-values under the alternative hypotheses can be approximated by Beta distributions.
2. The power of the tests are good, so that the mean p-values are much less than 1 under the alternative hypotheses.
3. The model weights account for the prior model probabilities and the mean p-values under the alternatives.

The construction of a Bayes factor from the HMP proceeds as follows. Since the p-value can be thought of as a low-dimensional summary of the full data, a Bayes factor can be defined as

$$\text{BF}_i = \frac{f_{A_i}(p_i)}{f_{O_i}(p_i)}, \qquad [48]$$

where $f_{O_i}(p)$ and $f_{A_i}(p)$ represent the probability density functions of the p-value under the null and alternative hypotheses respectively. By definition, the p-value follows a Uniform$(0,1)$ distribution under the null, so $f_{O_i}(p_i)=1$. Sellke, Bayarri and Berger (25) considered a Beta$(\xi,1)$ distribution for the p-value under the alternative, with $0<\xi\leq 1$, so the Bayes factor can be written

$$\text{BF}_i = \xi_i\, p_i^{\xi_i-1}. \qquad [49]$$

This would imply that the power of the hypothesis test has the form

$$\begin{aligned}\Pr(p_i<\alpha\, w_i\|A_i) &= \int_0^{\alpha\, w_i} f_{A_i}(p)dp \\ &= (\alpha\, w_i)^{\xi_i}\end{aligned} \qquad [50]$$

and the mean p-value under the alternative hypothesis would be

$$\begin{aligned}\mathbb{E}(p_i|A_i) &= \frac{\xi_i}{1+\xi_i} \\ &\approx \xi_i \qquad \text{if } \xi_i \ll 1. \end{aligned} \quad [51]$$

The model-averaged Bayes factor for the set of alternative hypotheses $\mathcal{R}$ versus the common null hypothesis would then be

$$\begin{aligned}\mathrm{BF}_{\mathcal{R}} &= \frac{\sum_{i\in\mathcal{R}} \mu_i \,\mathrm{BF}_i}{\sum_{i\in\mathcal{R}} \mu_i} \\ &= \frac{\sum_{i\in\mathcal{R}} \mu_i \,\xi_i \,p_i^{\xi_i-1}}{\sum_{i\in\mathcal{R}} \mu_i} \\ &\approx \frac{\sum_{i\in\mathcal{R}} \mu_i \,\xi_i \,p_i^{-1}}{\sum_{i\in\mathcal{R}} \mu_i} \qquad \text{if } \xi_i \ll 1 \\ &= \frac{\xi_{\mathcal{R}}}{\overset{\circ}{p}_{\mathcal{R}}} \end{aligned} \quad [52]$$

where

- μ_i is the prior probability of alternative hypothesis $\mathcal{M}_i$, conditional on rejection of the null $\mathcal{M}_0$, so that $\sum_{i=1}^{L} \mu_i = 1$.
- $w_i = \mu_i \xi_i / \sum_{j=1}^{L} \mu_j \xi_j$ is the weight for alternative hypothesis $\mathcal{M}_i$ in the HMP, so that $\sum_{i=1}^{L} w_i = 1$.
- $\xi_{\mathcal{R}} = \sum_{i\in\mathcal{R}} \mu_i \xi_i / \sum_{i\in\mathcal{R}} \mu_i$ is the mean p-value under the alternative hypotheses in set $\mathcal{R}$.
- $\bar{\xi} = \sum_{i=1}^{L} \mu_i \xi_i$ is the mean p-value averaged across all alternative hypotheses.

An important point to note is that more powerful tests are *penalized* in the calculation of the Bayes factor because the model weight w_i incorporates ξ_i, the mean p-value under the alternative, which is smaller for more powerful tests. The same weighting is obtained by optimizing the weights to maximize the frequentist power of the HMP (see below).

The Bayesian interpretation of the HMP allows posterior model probabilities to be calculated. For example, in the event that the null hypothesis is rejected, the posterior probability of hypothesis $\mathcal{M}_i$ can be computed as

$$\begin{aligned}\Pr(\mathcal{M}_i|\text{Data}) &= \frac{\mu_i \mathrm{BF}_i}{\sum_{j=1}^{L} \mu_j \mathrm{BF}_j} \\ &\approx \frac{w_i}{p_i} \overset{\circ}{p}. \end{aligned} \quad [53]$$

with $w_i = \mu_i \xi_i / \sum_{j=1}^{L} \mu_j \xi_j$. In this way, $100(1-\alpha)\%$ credibility intervals can be constructed by taking the smallest group of alternative hypotheses whose posterior probabilities, conditional on rejection of the null hypothesis, sum to at least $1-\alpha$. The harmonic mean p-value can also be written in the form of a Bayesian Information Criterion (26, 27) as

$$-2\log \mathrm{BF}_{\mathcal{R}} \approx 2\log \overset{\circ}{p}_{\mathcal{R}} - 2\log \xi_{\mathcal{R}}. \quad [54]$$

As above, this interpretation assumes that the weights are proportional to the product of the prior model probabilities and the mean p-values under the alternatives: $w_i \propto \mu_i \,\xi_i$. The disadvantage of this BIC is that the prior model probabilities and mean p-values under the alternatives need specifying. Instead, comparison of the posterior model probabilities (Equation 53) permits direct specification of only the model weights. Whether comparing BICs or posterior model probabilities for model selection, the same data and the same null hypothesis must be used in the calculation of every p-value. (This latter requirement is not restrictive if a sufficiently simple null hypothesis can be defined so that it is nested in every alternative hypothesis.)

Practical questions concerning the use of the HMP as an approximate Bayes factor or BIC are

1. For what values of ξ, the mean p-value under the alternative, is the approximation reasonable?

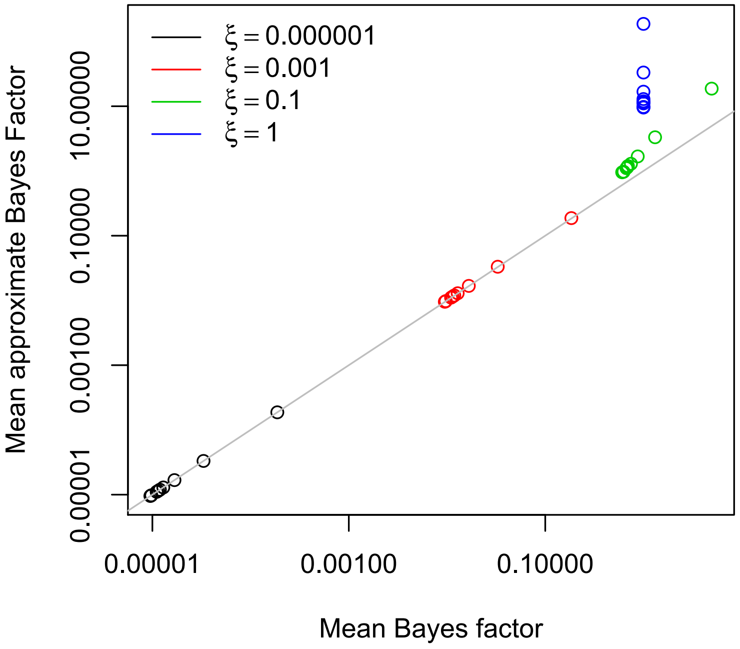

Fig. S9. Accuracy of the interpretation of the HMP as a Bayes factor in relation to the mean p-value under the alternative hypothesis, ξ. For values of ξ ranging from one to one millionth, 10,000 p-values were simulated under the null hypothesis, i.e. from a Uniform$(0, 1)$ distribution, and the Bayes factor and approximate Bayes factor (Equation 52) calculated. Both are shown on a logarithmic scale. For points lying on the grey line, the Bayes factor and approximate Bayes factor are identical. This shows that accuracy is reasonable for $\xi = 0.1$ and very good for smaller values of ξ.

2. What are the values of ξ?

3. What does use of equal prior weights imply?

Figure S9 addresses the first of these questions, showing that when the mean p-value under the alternative is $\xi = 0.1$, the approximation is already reasonably accurate, and for values below this it is very good. For context, when $\xi = 0.1$, the power is $\alpha^\xi = 0.74$ for the conventional significance threshold of $\alpha = 0.05$. This reiterates the requirement that the power needs to be good to interpret the HMP directly as a Bayes factor.

The question of what values the ξ_is take requires an assumption about the value or distribution of values of the parameters of the alternative hypothesis. Usually power is specified *a priori*, so this assumption amounts to a prior distribution, based on which power can be computed using a standard formula or numerically, for example by simulation. The prior distribution used for calculating power can be obtained in different ways, for example using previous data or by specifying a 'subjective' prior. Methods that specify an 'objective' prior that is improper cannot be used for Bayesian hypothesis testing nor for interpreting the HMP as a Bayes factor because, in the latter case, ξ would be undefined.

To take the multiple regression model used in the GWAS as an example, the power of each test can be calculated as follows. Suppose that only biallelic variants are considered, and numerically encoded in an $n \times L$ matrix G as 0 (common allele) or 1 (rare allele). Suppose that the vector y records the phenotypes and X is an $n \times c$ matrix of covariates, the first column of which is a vector of ones, corresponding to the intercept term, and the last column of which contains the genotypes from $G_{\cdot i}$. The other columns contain any other relevant covariates. The maximum likelihood estimate of the effect of genetic variant $G_{\cdot i}$ on the phenotype y is then

$$\hat{\beta}_i = \left\{ \left(X'X\right)^{-1} X'y \right\}_c \qquad [55]$$

and the variance of the estimator is

$$\begin{aligned} V\left(\hat{\beta}_i\right) &= \left\{ \left(X'X\right)^{-1} X'V\left(y\right) X \left(X'X\right)^{-1} \right\}_{c\,c} \\ &= \sigma_\epsilon^2 \left(X'X\right)^{-1}_{c\,c} \qquad [56] \end{aligned}$$

For large samples the test statistic

$$S_i = \frac{\hat{\beta}_i - \beta_0}{\sqrt{V\left(\hat{\beta}_i\right)}} \qquad [57]$$

follows a Normal$(0,1)$ distribution under the null hypothesis $\mathcal{M}_0$, in which $\beta_i = \beta_0$. If the parameter takes the value $\beta_i = \beta_A$ under alternative hypothesis $\mathcal{M}_i$ and *a priori* it is assumed that $\beta_A - \beta_0 \sim$ Normal$(0, \sigma_\beta^2)$, then the test statistic S_i follows a Normal$(0, 1 + \sigma_\beta^2/V(\hat{\beta}_i))$ distribution under the alternative hypothesis $\mathcal{M}_i$. The power of the test is

$$\begin{aligned}\Pr\left(p_i < \alpha|\mathcal{M}_i\right) &= \Pr\left(S_i^2 > Q_{\chi_1^2}(1-\alpha)|\mathcal{M}_i\right) \\ &= \Pr\left(\chi_1^2 > \frac{Q_{\chi_1^2}(1-\alpha)}{1+\sigma_\beta^2/V(\hat{\beta}_i)}\right) \qquad [58]\end{aligned}$$

where $Q_{\chi_1^2}$ is the quantile function of a χ^2 distribution with one degree of freedom. The mean p-value under $\mathcal{M}_i$ is

$$\mathbb{E}(p_i|\mathcal{M}_i) = 1 - \int_0^1 \Pr\left(p_i < \alpha|\mathcal{M}_i\right) \mathrm{d}\alpha \qquad [59]$$

which can be computed numerically for any given value of $\sigma_\beta^2/\sigma_\epsilon^2$ to provide $\xi_i = \mathbb{E}(p_i|\mathcal{M}_i)/(1-\mathbb{E}(p_i|\mathcal{M}_i))$. Thus power is computable by making a prior assumption about the relative magnitude of the genetic effects on the phenotype compared to the unexplained variance of the phenotype. If the ratio $\sigma_\beta^2/\sigma_\epsilon^2$ is assumed constant over all genetic variants, then power will be greater when $(X'X)^{-1}_{c\,c}$ is smaller.

The choice of $\sigma_\beta^2/\sigma_\epsilon^2$ could be based on prior information or subjective belief. An alternative approach is to find an 'objective' way to choose the prior. One objective could be to ensure that the condition $\xi_i \ll 1$ is met for all tests, and thereby validate the approximation of the Bayes factor from the HMP (Equation 52). Thus, the desired *power* could be specified *a priori*, and the value of $\sigma_\beta^2/\sigma_\epsilon^2$ required to achieve that power calculated per test.

If all tests were assumed to attain the same power, that would imply larger effects are expected *a priori* for tests with inherently lower power. This is a defensible approach because it effectively *penalizes* inherently lower-powered tests by requiring a higher standard of evidence, in terms of effect size, for the rejection of the null hypothesis. The penalty is imposed through the ξ_i term in the Bayes factor, which is smaller for tests that are assumed to be more powerful. In the context of GWAS for human disease, such priors are common and are advocated on the basis that risk alleles of larger effect would be maintained at lower frequency by natural selection, an assumption that can be tested in practice (28). Therefore the use of equal weights can be motivated by a uniform prior over the model probabilities for the alternative hypotheses, in conjunction with a prior that expects larger effect sizes for alternative hypotheses whose tests are inherently less powerful.

Optimal weights for the most powerful HMP. Interestingly, the weights suggested by the Bayesian interpretation of the HMP can be motivated by an argument based on optimizing the power of the HMP. The power of the HMP can be approximated roughly as

$$\begin{aligned}\Pr\left(\overset{\circ}{p} < \alpha|\mathcal{M}_A\right) &= \sum_{i=1}^{L} \Pr(\mathcal{M}_i|\mathcal{M}_A) \Pr\left(\overset{\circ}{p} < \alpha|\mathcal{M}_i\right) \\ &\approx \alpha + \sum_{i=1}^{L} \mu_i \Pr\left(p_i < \alpha\, w_i|\mathcal{M}_i\right) \\ &\approx \alpha + \sum_{i=1}^{L} \mu_i \left(\alpha\, w_i\right)^{\xi_i}. \qquad [60]\end{aligned}$$

This expression can be maximized subject to the constraint that $\sum_{i=1}^{L} w_i = 1$ by writing the Lagrangian function

$$\mathcal{L}\left(w, \lambda\right) = \alpha + \sum_{i=1}^{L} \mu_i \left(\alpha\, w_i\right)^{\xi_i} + \lambda \left[1 - \sum_{i=1}^{L} w_i\right]. \qquad [61]$$

Solving the derivative with respect to w_i gives

$$w_i^{1-\xi_i} = \frac{1}{\lambda}\mu_i\,\xi_i\,\alpha^{\xi_i} \qquad [62]$$

which for $\xi_i \ll 1$ is solved by

$$\begin{aligned} w_i &\approx \frac{1}{\lambda}\mu_i\,\xi_i\,\alpha^{\xi_i} \\ &\approx \frac{1}{\lambda}\mu_i\,\xi_i \end{aligned} \qquad [63]$$

where the constant λ imposes the constraint that $\sum_{i=1}^{L} w_i = 1$. So power appears to be optimized in the frequentist setting by (i) up-weighting the inverse p-values of alternative hypotheses that are more probable *a priori* and (ii) down-weighting the inverse p-values of more powerful tests, recapitulating the weights required for a Bayesian interpretation of the HMP.

D. Relaxing significance thresholds when multiple alternative hypotheses are true. Some procedures for controlling the FDR explicitly estimate the proportion of alternative hypotheses that are true, reducing the stringency of the threshold for calling a test significant when a larger proportion of alternatives are estimated to be true (see e.g. refs (29–33)). The question arises whether similar reasoning can be used to motivate a relaxation of the significance threshold in the model-averaging setting.

Consider, for example, a GWAS in bacteria for a trait of interest, such as antimicrobial resistance (34, 35). Antimicrobial resistance is often influenced by multiple loci, but the tests at different loci cannot be treated as independent because of strong dependency (i.e. linkage disequilibrium) between loci caused by relatively low rates of genetic exchange and strong structuring of populations into sub-populations or 'strains' (35). To formally test for the involvement of multiple loci in the trait, it would be necessary to fit all higher-order models involving two or more loci. However, to test for the involvement of K loci would involve $\binom{L}{K}$ tests, which quickly becomes very large even for modest K.

Even in human GWAS, tests at different loci can never be truly independent because the same outcomes (the phenotypes) are shared between analyses. However, it is often considered appropriate to test associations between multiple loci and a single trait as if they were independent, particularly for loci that are physically distant or on different chromosomes (i.e. unlinked loci), and it is in this context that FDR arguments are applied to human GWAS (29). Since the control of the FDR in human GWAS carries an explicit assumption that multiple loci might be associated with the trait, it therefore carries an *implicit* assumption that the signal of association at any individual locus tested alone is in some sense averaged over all the other loci which might also be associated. In other words, the so-called 'marginal' (i.e. single locus) test for the association is implicitly assumed to approximate an equivalent model-averaged test.

Stating this explicitly, arguments in favour of controlling the FDR in GWAS assume, perhaps optimistically, that the Bayes factor or p-value for the marginal test approximates a model-averaged Bayes factor or p-value. Let $\mathcal{M}_{i\bullet}$ denote the set of all models involving locus i and $(K-1)$ other loci, of which there are $\binom{L-1}{K-1}$ combinations. The criterion for declaring a significant association involving a particular locus i, according to this approximation, is

Bayesian	HMP
$\frac{\Pr(\mathcal{M}_{i\bullet}\|\mathbf{X})}{\Pr(\mathcal{M}_0\|\mathbf{X})} \approx \frac{\Pr(\mathcal{M}_{i\bullet})}{\Pr(\mathcal{M}_0)}\,\frac{\Pr(\mathbf{X}\|\mathcal{M}_i)}{\Pr(\mathbf{X}\|\mathcal{M}_0)} \geq \frac{1-\alpha}{\alpha}$	$\alpha_{\binom{L-1}{K-1}}\,w_{i\bullet} \geq \mathring{p}_{i\bullet} \approx p_i$
$\frac{\alpha\,(1-\mu_0)}{\mu_0\,(1-\alpha)}\,\mu_{i\bullet}\,\mathrm{BF}_i \geq 1$	$\alpha_{\binom{L-1}{K-1}}\,w_{i\bullet}\,b_i \geq 1$

In the case of equal weights among the $\binom{L}{K}$ hypotheses, then $w_{i\bullet} = \binom{L-1}{K-1}/\binom{L}{K} = \frac{K}{L}$, which is a factor K times bigger than $w_i = \frac{1}{L}$. Therefore this approximation motivates the relaxation of the stringency of the significance threshold by a factor K, where K is the number of loci involved in the trait, so the threshold is now a function of the *proportion* of alternative hypotheses expected to be true, rather than simply the number of tests. The obvious problem is that either a strong assumption regarding the true value of K has to be made or K has to be estimated somehow, e.g. by explicitly evaluating the higher-order tests over all sets of K loci for different values of K. If this latter procedure were computationally feasible, the approximation would be unnecessary. Nevertheless, the effective relaxation of the multiple testing threshold would still be gained because $w_{i\bullet}$ would inevitably increase if more loci were involved in the trait.

6. GWAS of neuroticism

I downloaded the p-values for 6 524 432 directly assayed and imputed variants arising from a meta-analysis of neuroticism in 170 911 individuals (36), one of many human GWAS catalogued by LD Hub (37), and whose summary statistics are freely publicly available (http://ssgac.org/documents/Neuroticism_Full.txt.gz). These p-values were calculated from a meta-analysis of numerous cohorts using METAL (38). METAL conducts a z-test which is analogous to a likelihood ratio test for whether the effect of each variant on the trait is different from zero. The common null hypothesis is that no variant has an effect. I defined overlapping sliding windows of various sizes: 10kb, 100kb, 1000kb, 10Mb, entire chromosomes and the whole genome. I computed the HMP (Equation 8) and corresponding p-value (Equation 4) for each window. I computed adjusted values by dividing the HMP and corresponding p-value for the region $\mathcal{R}$ spanned by each window by $w_{\mathcal{R}}$, the sum of the weights across all variants within the window. Since I assumed equal weights across variants, $w_{\mathcal{R}}$ equalled the proportion of all variants within the window. I then compared the adjusted HMPs and p-values directly to the significance threshold $\alpha = 0.05$.

7. GWAS of HCV pre-treatment viral load

I applied to the STOP-HCV consortium (http://www.stop-hcv.ox.ac.uk/data-access) for access to the 399 420 human genotypes and 410 viral polyprotein sequences from a GWAS of pre-treatment viral load in 410 white European patients with HCV genotype 3a (39). I imputed missing base calls and indels in the aligned HCV nucleotide sequences using ClonalFrameML (40) having estimated a maximum likelihood phylogeny using PhyML (41). I converted to a codon alignment and identified 827 variable codons in which the frequency of the consensus codon was less than 95%. I conducted 330 320 340 tests of association between $\log_{10}$ pre-treatment viral load and the interaction between each pair of human and virus variant. I fitted a linear model with the following factors and covariates: human variant, virus variant, an interaction between human variant and virus variant, and, following (39) , the leading three principal components (PCs) of human genetic variation, the leading ten PCs of virus genetic variation, patient sex and an indicator of sustained virological response (SVR). I assumed codominance between human alleles. Each p-value was produced by a likelihood ratio test between the fitted model and the common null hypothesis in which only the three human PCs, ten virus PCs, sex and SVR were included.

For each human variant, I computed the HMP (Equation 8) by averaging across all $L_P = 827$ interactions involving that variant. Conversely, I computed the HMP for each virus variant by averaging across all $L_H = 399\,420$ interactions involving that variant. I computed a p-value (Equation 4) for every HMP. I compared the p-value for each subset of hypotheses $\mathcal{R}$ against a threshold $\alpha\, w_{\mathcal{R}}$, where $w_{\mathcal{R}}$ was the sum of weights of all alternative hypotheses in subset $\mathcal{R}$. Since I assumed equal weights across all pairs of variants, the thresholds for the 330 320 340 tests of association, the 399 420 model-averaged p-values per human variant and the 827 model-averaged p-values per virus variant were $\alpha/(L_H L_P) = 1.5 \times 10^{-10}$, $\alpha/L_H = 1.3 \times 10^{-7}$ and $\alpha/L_P = 6.0 \times 10^{-5}$ respectively.

To quantify the relative strength of evidence among the alternative hypotheses, having rejected the common null hypothesis, I calculated the approximate 'post-data' or 'posterior' probability of each alternative particular hypothesis $\mathcal{M}_i$. This was possible because I treated the model formally as a random effect in which the model weights take into account a 'pre-data' or 'prior' probability distribution and the power of each test, and is analogous to, e.g., posterior decoding in a hidden Markov model:

$$\Pr(\mathcal{M} = \mathcal{M}_i | \mathbf{X}, \mathcal{M} \neq \mathcal{M}_0) \quad \approx \quad \frac{w_i/p_i}{\sum_{j=1}^{L} w_j/p_j}. \qquad [64]$$

This approximation is justified in section §5. I took $L = (L_H\, L_P)$, the p_is from each of the L likelihood ratio tests and equal weights $w_i = 1/(L_H L_P)$.

8. SI references

1. Wilks SS (1938) The large-sample distribution of the likelihood ratio for testing composite hypotheses. *Annals of Mathematical Statistics* 9(1):60–62.
2. Uchaikin VV, Zolotarev VM (1999) *Chance and stability: Stable distributions and their applications.* (Walter de Gruyter).
3. Karamata J (1933) Sur un mode de croissance régulière. théorèmes fondamentaux. *Bull. Soc. Math. France* 61:55–62.
4. Mikosch T (1999) *Regular variation, subexponentiality and their applications in probability theory.* (Eindhoven University of Technology).
5. Zaliapin I, Kagan YY, Schoenberg FP (2005) Approximating the distribution of pareto sums. *Pure and Applied Geophysics* 162(6):1187–1228.
6. Rimmer RH, Nolan JP (2005) Stable distributions in mathematica. *Mathematica Journal* 9(4):776–789.
7. Robinson G (2012) *FMStable: Finite Moment Stable Distributions.* R package version 0.1-2.
8. Landau LD (1944) On the energy loss of fast particles by ionization. *Journal of Physics U.S.S.R.* 8(4):201–205.
9. Landau LD (1965) On the energy loss of fast particles by ionization in *Collected papers of L. D. Landau*, ed. ter Haar D. (Pergamon Press, Oxford), pp. 417–424.
10. Uchaikin VV, Topchii VA (1978) Stable law with index $\alpha = 1$ in the problem of fluctuations of ionization losses of charged particles. *Izvestiya Vysshikh Uchebnykh Zavedenii, Fizika* 4:60–64.
11. Uchaikin VV, Topchii VA (1978) Stable law with index $\alpha = 1$ in the problem of fluctuations of ionization losses of charged particles. *Soviet Physics Journal* 21(4):459–462.
12. Kölbig KS, Schorr B (1984) A program package for the Landau distribution. *Computer Physics Communications* 31:97–111.
13. Bartkiewicz K, Jakubowski A, Mikosch T, Wintenberger O (2011) Stable limits for sums of dependent infinite variance random variables. *Probability Theory and Related Fields* 150(3):337–372.

14. Marcus R, Eric P, Gabriel KR (1976) On closed testing procedures with special reference to ordered analysis of variance. *Biometrika* 63(3):655–660.
15. Simes RJ (1986) An improved Bonferroni procedure for multiple tests of significance. *Biometrika* 73(3):751–754.
16. Hochberg Y, Liberman U (1994) An extended simes' test. *Statistics & Probability Letters* 21(2):101–105.
17. Ramdas A, Barber RF, Wainwright MJ, Jordan MI (2017) A unified treatment of multiple testing with prior knowledge. *arXiv preprint arXiv:1703.06222*.
18. Fisher RA (1934) *Statistical Methods for Research Workers*. (Oliver and Boyd, Edinburgh), Fifth edition.
19. Loughin TM (2004) A systematic comparison of methods for combining p-values from independent tests. *Computational Statistics & Data Analysis* 47(3):467–485.
20. Lancaster H (1961) The combination of probabilities: an application of orthonormal functions. *Australian & New Zealand Journal of Statistics* 3(1):20–33.
21. Liptak T (1958) On the combination of independent tests. *Magyar Tud Akad Mat Kutato Int Kozl* 3:171–197.
22. Edgington ES (1972) An additive method for combining probability values from independent experiments. *The Journal of Psychology* 80(2):351–363.
23. Mudholkar GS, George E (1979) The logic method for combining probabilities in *Symposium on Optimizing Methods in Statistics*, ed. Rustagi J. (Academic Press, New York), pp. 345–366.
24. Benjamini Y, Hochberg Y (1995) Controlling the false discovery rate: a practical and powerful approach to multiple testing. *Journal of the Royal Statistical Society. Series B* 57(1):289–300.
25. Sellke T, Bayarri MJ, Berger JO (2001) Calibration of p values for testing precise null hypotheses. *The American Statistician* 55(1):62–71.
26. Schwarz G, , et al. (1978) Estimating the dimension of a model. *Annals of Statistics* 6(2):461–464.
27. Raftery AE (1996) Approximate bayes factors and accounting for model uncertainty in generalised linear models. *Biometrika* 83(2):251–266.
28. Speed D, et al. (2017) Reevaluation of SNP heritability in complex human traits. *Nature Genetics* 49:986–992.
29. Stephens M, Balding DJ (2009) Bayesian statistical methods for genetic association studies. *Nature Reviews Genetics* 10(10):681.
30. Efron B, Tibshirani R, Storey JD, Tusher V (2001) Empirical bayes analysis of a microarray experiment. *Journal of the American Statistical Association* 96(456):1151–1160.
31. Storey JD, Tibshirani R (2003) Statistical significance for genomewide studies. *Proceedings of the National Academy of Sciences* 100(16):9440–9445.
32. Storey JD (2002) A direct approach to false discovery rates. *Journal of the Royal Statistical Society: Series B* 64(3):479–498.
33. Stephens M (2016) False discovery rates: a new deal. *Biostatistics* 18(2):275–294.
34. Chewapreecha C, et al. (2014) Comprehensive identification of single nucleotide polymorphisms associated with beta-lactam resistance within pneumococcal mosaic genes. *PLoS Genetics* 10(8):e1004547.
35. Earle SG, et al. (2016) Identifying lineage effects when controlling for population structure improves power in bacterial association studies. *Nature Microbiology* 1:16041.
36. Okbay A, et al. (2016) Genetic variants associated with subjective well-being, depressive symptoms, and neuroticism identified through genome-wide analyses. *Nature Genetics* 48(6):624–633.
37. Zheng J, et al. (2017) Ld hub: a centralized database and web interface to perform ld score regression that maximizes the potential of summary level GWAS data for SNP heritability and genetic correlation analysis. *Bioinformatics* 33(2):272–279.
38. Willer CJ, Li Y, Abecasis GR (2010) Metal: fast and efficient meta-analysis of genomewide association scans. *Bioinformatics* 26(17):2190–2191.
39. Ansari MA, et al. (2017) Genome-to-genome analysis highlights the effect of the human innate and adaptive immune systems on the hepatitis c virus. *Nature Genetics* 49(5):666–673.
40. Didelot X, Wilson DJ (2015) Clonalframeml: efficient inference of recombination in whole bacterial genomes. *PLoS Computational Biology* 11(2):e1004041.
41. Guindon S, et al. (2010) New algorithms and methods to estimate maximum-likelihood phylogenies: assessing the performance of phyml 3.0. *Systematic Biology* 59(3):307–321.
